# Why do proteins aggregate? “Intrinsically insoluble proteins” and “dark mediators” revealed by studies on “insoluble proteins” solubilized in pure water

**DOI:** 10.12688/f1000research.2-94.v1

**Published:** 2013-03-22

**Authors:** Jianxing Song

**Affiliations:** 1 Department of Biological Sciences, Faculty of Science, National University of Singapore, Singapore, 119260, Singapore; 2 Department of Biochemistry, Yong Loo Lin School of Medicine, National University of Singapore, Singapore, 119260, Singapore

## Abstract

In 2008, I reviewed and proposed a model for our discovery in 2005 that unrefoldable and insoluble proteins could in fact be solubilized in unsalted water. Since then, this discovery has offered us and other groups a powerful tool to characterize insoluble proteins, and we have further addressed several fundamental and disease-relevant issues associated with this discovery. Here I review these results, which are conceptualized into several novel scenarios. 1) Unlike 'misfolded proteins', which still retain the capacity to fold into well-defined structures but are misled to 'off-pathway' aggregation, unrefoldable and insoluble proteins completely lack this ability and will unavoidably aggregate in vivo with ~150 mM ions, thus designated as 'intrinsically insoluble proteins (IIPs)' here. IIPs may largely account for the 'wastefully synthesized' DRiPs identified in human cells. 2) The fact that IIPs including membrane proteins are all soluble in unsalted water, but get aggregated upon being exposed to ions, logically suggests that ions existing in the background play a central role in mediating protein aggregation, thus acting as 'dark mediators'. Our study with 14 salts confirms that IIPs lack the capacity to fold into any well-defined structures. We uncover that salts modulate protein dynamics and anions bind proteins with high selectivity and affinity, which is surprisingly masked by pre-existing ions. Accordingly, I modified my previous model. 3) Insoluble proteins interact with lipids to different degrees. Remarkably, an ALS-causing P56S mutation transforms the β-sandwich MSP domain into a helical integral membrane protein. Consequently, the number of membrane-interacting proteins might be much larger than currently recognized. To attack biological membranes may represent a common mechanism by which aggregated proteins initiate human diseases. 4) Our discovery also implies a solution to the 'chicken-and-egg paradox' for the origin of primitive membranes embedded with integral membrane proteins, if proteins originally emerged in unsalted prebiotic media.

## Introduction

### Self-assembly and protein folding

Life represents extremely unique systems that supposedly emerged from inanimate nature, but its origin remains a great mystery. As formulated by Schrödinger in 1944 in his book "What is Life?" ^1^, life is characteristic of two fundamental processes; 'order from order' and 'order from disorder'. Schrödinger predicted that the gene controlling the 'order from order' process in a species was an aperiodic crystal, which was uncovered to be DNA a decade later by Watson and Crick. So far, the mechanisms, functions and structures of life associated with 'order from order' have been extensively unraveled. On the other hand, another process for achieving 'order from disorder' that drove chemical evolution and led to the emergence of life from primitive physical systems has remained a subject of continuing debate and uncertainty ^1^.

As self-assembly occurs at all levels of living systems, life appears to defy the second law of thermodynamics, which states that any spontaneous process is associated with the overall increase of entropy. In this regard, Schrödinger theorized that life, contrary to the general tendency dictated by the second law of thermodynamics, decreases or maintains its entropy by feeding on negative entropy. When Lovelock was asked in 1964 what he would do to look for life on Mars, he replied: "I’d look for an entropy reduction, since this must be a general characteristic of life" ^2^. Later Prigogine proposed that living systems are dissipative structures existing far from equilibrium, into which importation and dissipation of energy could reverse the maximization of entropy rule imposed by the second law of thermodynamics ^3^. Recently the self-organization in living systems has been proposed as a nature process resulting from the consumption of free energy ^4, 5^.

Proteins are the most important functional players that implement difficult, but essential tasks in living cells. They are linear heteropolymers composed of 20 common α-amino acids, which amazingly are all in L-mirror-image ^6, 7^. Intriguingly, a recent analysis indicated that the human genome has ~20,687 protein-coding genes ^8^, which are remarkably smaller than those of many less complex organisms, such as the roundworm and the fruit fly. This may result from the extensive use of alternative splicing in humans, which provides the ability to build a very large number of modular proteins through the selective incorporation of exons.

Proteins also represent one of the best examples to illustrate the self-assembly of biomolecules. Remarkably, a portion of proteins will spontaneously self-organize into unique three-dimensional structures via protein folding processes ^9– 17^. It is widely recognized that the folding of cytosolic proteins is mainly resulting from solvophobic interactions of polar water molecules with the hydrophobic side chains of proteins. As a result, in the three-dimensional structure of a well-folded cytosolic protein, more than 80% of hydrophobic side-chains are buried in the internal core, thus being shielded from water, while most hydrophilic residues are exposed to polar water molecules. On the other hand, however, it has been now widely realized that many proteins are fully functional, but lack well-defined structures, and are thus called intrinsically unstructured/disordered proteins (IUPs). It has been estimated that ~50% of cellular proteomes encode proteins with long intrinsically unstructured domains/fragments ^18– 23^. Interestingly, a recent study indicates that there is a sharp increase in disorder associated with the transition from prokaryotic to eukaryotic cells ^23^. Intrinsically unstructured proteins have been found to be significantly lacking in bulky hydrophobic (Ile, Leu, and Val) and aromatic (Trp, Tyr, and Phe) residues but dramatically enriched in polar (Arg, Gly, Gln, Ser, Pro, Glu, and Lys) and structure-breaking (Gly and Pro) residues ^18– 23^.

Modern life forms all begin with cells, which are separated from the external environment by a surface membrane, called the plasma membrane. Furthermore, eukaryotic cells have extensive internal membranes that further subdivide the cell into various compartments, which are all filled with watery media. Life on earth absolutely depends on water, which constitutes 70–80%, by weight, of most cells. Therefore, water is regarded as the ‘matrix of life’ which not only provides a passive scaffold, but also has many active roles in molecular biology ^24– 30^. The absolutely essential role of water for life is associated with its unique combination of many different properties, which are believed to be irreplaceable by another single molecule system. For example, water exhibits a solvophobic effect, which thus renders hydrophobic effects to play a dominant role in all self-organizing processes in biology ^12– 17^.

On the other hand, in addition to providing a barrier to prevent the free flow of molecules in and out of cells, biological membrane systems appear also to provide a hydrophobic environment in cells. All biological membranes are composed of lipid molecules, consisting of a hydrophilic headgroup, and one or more hydrophobic fatty acid hydrocarbon tails. Due to their amphiphilic nature, lipid molecules spontaneously self-organize into bilayers in cells, consisting of two sheets of lipids with their hydrophobic tails facing each other to form the hydrophobic core, which is shielded from the aqueous surroundings by a polar interface consisting of the hydrophilic headgroups. There is no sharp border between the hydrophobic core and the surrounding water, as this interface region has gradually changing hydrophobicity ^31^. In 1992, Wiener and White determined the structure of a bilayer, in which the hydrophobic core is about 30 Å thick, while the interface region extended about 15 Å on either side ^32^. As a consequence, it is anticipated that biological membranes also provide a non-polar phase to accommodate hydrophobic proteins. Indeed, it has been estimated that more than 30% of proteins are located in membrane environments, which are highly hydrophobic ^33– 35^. Nevertheless, it appears to be much more complex for the folding of membrane proteins as lipid bilayers show large variations in density and polarity on a nanometer scale. While the center is highly hydrophobic and significantly disordered, the surfaces consist of diverse mixture of polar functional groups including the carbonyl and glycerol groups, which extensively interact with water molecules. As such, all attempts to use organic solvent to mimic membranes failed ^33– 42^.

### Protein aggregation and solubilizing 'insoluble proteins' with unsalted water

One intriguing phenomenon associated with proteins is their insolubility in aqueous buffers. Protein aggregation/insolubility is not only problematic for *in vitro* protein research and industry applications, but is commonly associated with a large spectrum of human disease, in particular neurodegenerative/aging diseases including Parkinson’s disease (PD), Alzheimer’s disease (AD), Huntington’s disease (HD), spinocerebellar ataxias (SCA), amyotrophic lateral sclerosis (ALS), aging and many others. These diseases have the common characteristic of the aggregation of disease-specific proteins and are thus called 'conformational diseases' ^43– 54^. Recently, protein aggregation has been increasingly revealed to be involved in other human diseases ^55, 56^, and also has important implications in fields other than biology, including nanotechnology and material science ^57^.

Interestingly, both genetic mutations (familiar) or environmental insults (sporadic) can trigger protein aggregation diseases, implying that a common mechanism may exist to link these clinically distinct diseases. It has been extensively thought that the aggregation of these proteins is due to misfolding. In other words, these proteins still have the intrinsic ability to fold into well-defined structures, but have been misled to aggregation through 'off-pathway' misfolding triggered by abnormal cellular and environmental factors. Indeed, it has been shown that all folded proteins could be induced to form amyloid aggregates *in vitro* by manipulating solution conditions ^45, 46^. Nevertheless, recent studies increasingly reveal that protein aggregation is not a rare event under abnormal conditions. Most unexpectedly, a recent study on human cells reveald that approximately 30% of cellular proteins are 'wastefully synthesized' ^58^. Immediately after their synthesis, these proteins get aggregated and are thus rapidly degraded by proteasomes ^58^. This strongly implies that even under normal cellular environments, aggregation is an evitable destination for a large portion of proteins. As accumulation of aggregated protein can dramatically perturb protein homeostasis and can lead to extensive cell and tissue damage through a variety of mechanisms, cells have developed various quality control systems in evolution to remove misfolded/aggregated proteins ^58– 65^.

So why do proteins become insoluble/aggregated? A complete answer to this question not only sheds light on the fundamental properties associated with proteins, but is also crucial to deciphering the mechanisms underlying a variety of human diseases as well as to further developing therapeutic strategies and agents. Certainly, some proteins become aggregated from well-folded soluble forms through 'off-pathway' misfolding, which can be significantly suppressed by cellular chaperone systems. However, there exist a portion of proteins which appear to be not refoldable and completely insoluble in various buffer conditions, as particularly demonstrated by the results from structural genomics projects ^66^. Previously, biophysical studies have been exhaustively conducted on misfolded proteins to understand the factors mediating their aggregation. In contrast, high-resolution studies on unrefoldable and insoluble proteins have not been possible due to the absence of a general method to solubilize these insoluble proteins without adding denaturants and detergents. Nevertheless, many disease-causing protein mutants appear to belong to this category, as exemplified by the ALS-causing P56S mutant of the VAPB-MSP domain ^67^. Because of this, a fundamental question remains unanswered as to by which degree these mutations alter the folding properties of these mutant proteins.

Marvelously, in 2005 we discovered that all 11 unrefoldable and insoluble proteins we had could be solubilized in unsalted water for high-resolution nuclear magnetic resonance (NMR) investigations ^68, 69^. The results led to the classification of these 'insoluble proteins' into three groups: group 1, with no secondary and tertiary structures; group 2, with only secondary structure but no tertiary packing; and group 3, with secondary structure and loose tertiary packing, like molten globule states. Remarkably, we found that all these insoluble proteins lack tight tertiary packing. Therefore, I have proposed that unrefoldable and insoluble proteins may lack the intrinsic ability to fold into well-defined structures, thus with many hydrophobic side chains exposed to the polar water molecules. As such, a very low ionic strength is sufficient to non-specifically screen out intrinsic repulsive interactions and, thus, trigger hydrophobic clustering/aggregation ^68^. In 2008, I further proposed a model to rationalize our discovery and explain why our results appear to be inconsistent with the well-established dogma about the effect of salt on protein solubility ^26^.

### Scope of this review

Our discovery led to the establishment of a general and powerful tool to characterize unrefoldable and insoluble proteins. Indeed, with this tool other groups have studied insoluble proteins, including those involved in biomineralization ^70– 76^. On the other hand, this also allows us to address several fundamental and disease-relevant issues associated with the discovery. Here I review the results of the studies based on our discovery. Analysis of these results in the relevant contexts leads to the conceptualization of several novel scenarios.

Firstly, we characterized solution conformations and dynamics of several unrefoldable and insoluble proteins including those with low complexity sequences, as well as resulting from the splicing variation and insertion/mutation on well-folded domains such as SH3 and MSP. The results uncover that unlike 'misfolded proteins', which still have the capacity to fold into uniquely well-defined structures, unrefoldable and insoluble proteins all lack this ability due to either low complexity sequences, splicing variation, or insertion/mutation in the well-folded domains. As a consequence, they will unavoidably aggregate *in vivo* with ~150 mM ion concentration, and are thus here designated as 'intrinsically insoluble proteins (IIPs)'.

Secondly we assessed the upper limit of our discovery with the 25-residue integral membrane peptide of the influzene M2 channel, one of the most hydrophobic protein sequences in nature. To our great surprise, it could also be solubilized in unsalted water without lipid molecules to form a highly helical conformation.

Thirdly, the fact that insoluble proteins including integral membrane proteins could all be solubilized in unsalted water, but become aggregated upon being exposed to salt ions logically suggests that salt ions play central roles in mediating protein aggregation, and are thus designated as 'dark mediators' here. To delineate the underlying mechanisms, we systematically assessed the effects of 14 salts with 8 anions on the conformation of unrefoldable and insoluble proteins dissolved in unsalted water. This led to the discovery that anions have asymmetric binding to both unstructured and well-folded proteins with high selectivity and affinity, which is surprisingly masked by pre-existing ions. Very recently, we discovered that different salts have very diverse effects on protein dynamics.

Fourthly, we have also tested whether insoluble proteins are generally capable of interacting with membranes. This leads to our discovery that the ALS-causing P56S mutation and the truncation in the VAPB-3 splicing variant transform the seven-strand immunoglobulin-like β-sandwich adopted by the native MSP domain into a helical structure with the majority of residues buried in membranes.

Fifthly, in light of our discovery, I hypothesize a solution to the 'chicken-and-egg' paradox for the origin of primitive cells embedded with integral membrane proteins if all proteins originally emerged in unsalted prebiotic oceans/media.

## Intrinsically insoluble proteins

### Proteins with low-complexity sequences are unstructured and insoluble

Due to low-complexity sequences, lacking of hydrophobic and aromatic and enrichment of polar residues, intrinsically unstructured proteins have been generally thought to be highly soluble in buffers. However, by solubilizing it in unsalted water, we have characterized a transcriptional activator, ApLLP, to be both insoluble and intrinsically unstructured ^22^.

**Figure 1.**
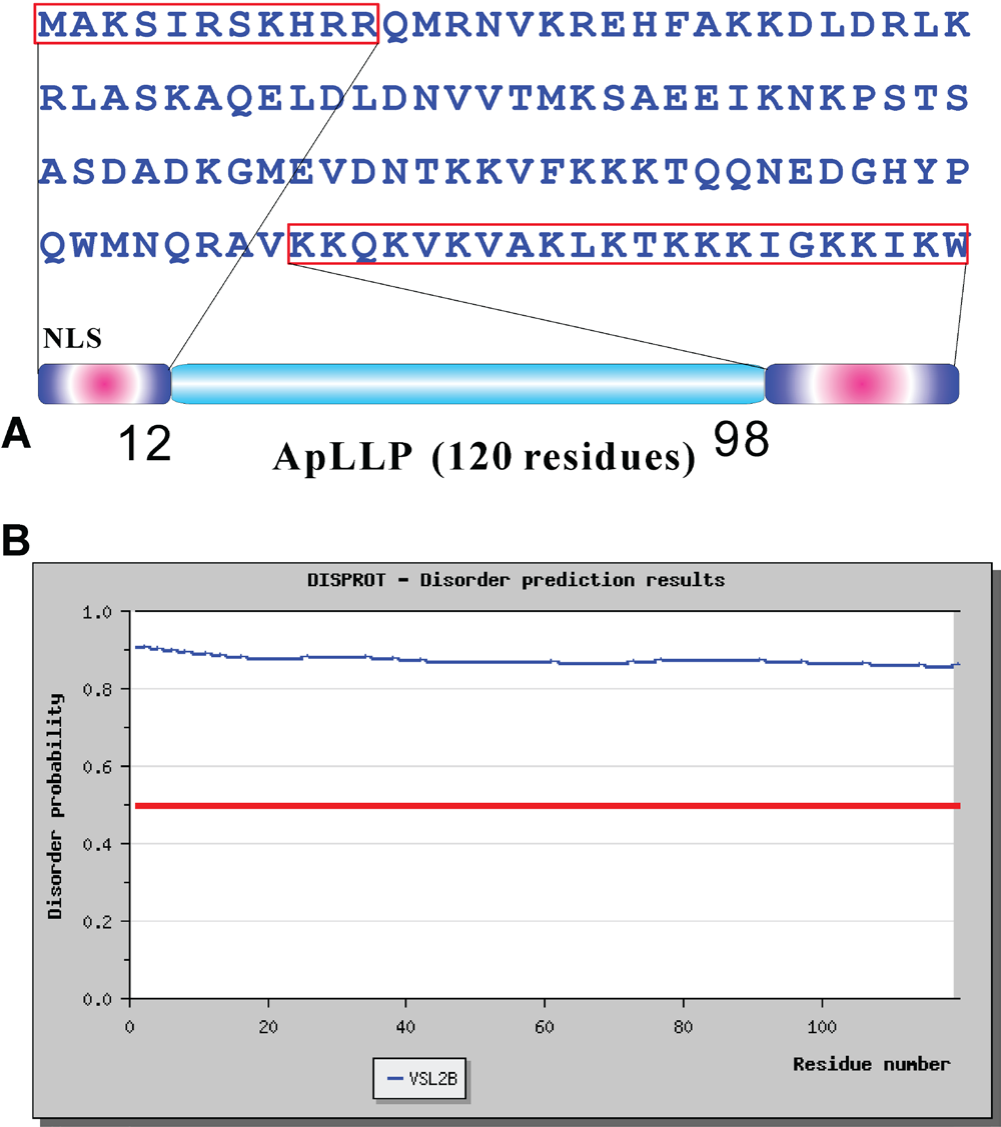
Sequence properties of ApLLP, a 120-residue *Aplysia* transcriptional activator. ( **A**) The sequence of ApLLP, with two Nuclear Localization Signals (NLS) boxed. ( **B**) Analysis results of the globularity and disordered regions of ApLLP by VSL2B.

The 120-residue ApLLP ( *Aplysia* LAPS18-like protein) is a transcription factor which is required for long-term synaptic facilitation in *Aplysia* neurons. As seen in Figure 1A, ApLLP is characteristic of a protein with low hydrophobicity, containing only 25.83% hydrophobic residues. Further assessment by DISPROT in Figure 1B also revealed that it has a disorder probability larger than 0.8 over the whole sequence, strongly suggesting that ApLLP is intrinsically unstructured.

**Figure 2.**
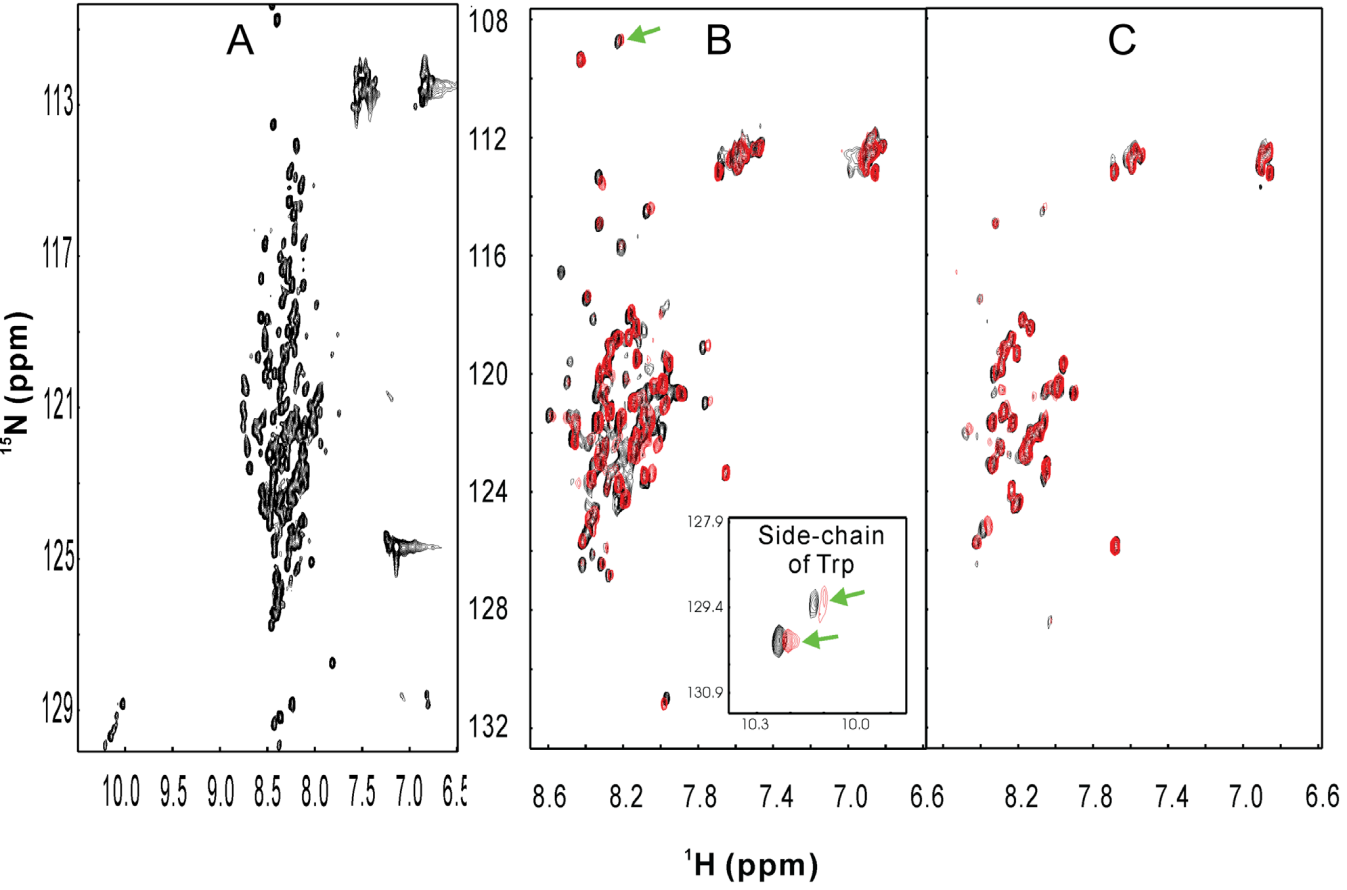
Conformational and binding properties of the full-length and two truncated ApLLP forms. ( **A**) ^1^H– ^15^N NMR HSQC spectrum of the ^15^N-labeled full-length ApLLP solubilized in unsalted water. ( **B–C**) Superimposition of the ^1^H– ^15^N NMR HSQC spectra of ApLLP-87 ( **B**) and ApLLP-55 ( **C**) in the absence and in the presence of the DNA fragment at a molar ratio of 1/2 (protein/DNA). Shifted HSQC peaks arising from the Gly backbone and Trp side-chain (inlet) only presented in ApLLP-87 are indicated by green arrows.

Intriguingly, despite its low hydrophobicity, ApLLP was highly insoluble in a variety of buffers tested, but could be solubilized in unsalted water at high concentrations (>200 µM). CD and NMR characterization clearly indicated that it was predominantly disordered without any stable secondary and tertiary structures ( Figure 2). Unexpectedly, if one or two nuclear localization signals located at N- and C-termini were removed, the truncated ApLLP suddenly became soluble in buffers such as 20 mM phosphate buffer. However, the two nuclear localization signal sequences have no detectable difference of hydrophobicity from the rest of the protein.

As demonstrated by NMR titrations in Figure 2A–2B, both truncated ApLLP forms were able to bind the CRE DNA element. However, even upon forming the ApLLP-DNA complex, ApLLP still appeared to be highly disordered, as evident from NMR and CD results ( Figure 2). As such, we had a very difficult time to get the results, eventually published ~2.5 years after the first submission, because some reviewers could not be convinced by two observations, 1) ApLLP is highly insoluble in buffer despite owning a highly degenerative sequence and with low hydrophobicity. 2) Even upon complexing with DNA, the intrinsically unstructured ApLLP failed to form a well-folded complex structure. Now, this phenomenon is starting to be recognized to exist in a large variety of protein-protein and protein-DNA complexes involved in intrinsically unstructured proteins, and thus designated as the 'fuzzy complex' ^73, 77– 80^.

Similar features have since been observed by another group on the basic helix-loop-helix motif (bHLH) region of a transcription factor NGN1 ^73^. It was found that "bHLHN was soluble in pure water at any concentration". On the other hand, upon binding to two DNA E-boxes, the protein also appeared to be highly disordered.

Remarkably, a group of proteins involved in biomineralization that are highly insoluble in buffers has also been demonstrated to be soluble in unsalted water at high protein concentrations that are suitable for detailed biophysical characterizations ^70– 72, 75, 76^. Biomineralization, which generates the hard tissues of living organisms, is a process under tight regulation by hormones, enzymes and various regulatory proteins. However, many of them have resisted structural characterization because of their high insolubility ^75, 76, 81– 83^. For example, the 172-residue porcine amelogenin, found in mammalian tooth enamel, is one of the most highly mineralized materials of vertebrates. This protein is essential for normal enamel development and undergoes self-association to form supramolecular assemblies under defined conditions in the laboratory ^70^. However, its aggregation could be suppressed in unsalted water and exhaustive biophysical studies including detailed assignments by three-dimensional NMR experiments revealed that it is intrinsically disordered with an extended configuration in the monomeric form ^70^.

Recently, a systematic bioinformatics study unraveled that all proteins in SwissProt that have been annotated for biomineralization show an extremely high level of predicted disorder (with a mean of 53%), which represents the most disordered class of the protein world. Furthermore, the same feature was associated with evolutionarily more distant proteins involved in the formation of the silica wall of marine diatoms and the shell of oysters and other mollusks. The general and very strong correlation between biomineralization and structural disorder has been proposed to indicate that controlled growth of the mineral phase in biology may only be achieved with the assistance of highly disordered proteins ^82^. In this regard, both structural disorder and high insolubility seem essential for their functions in coordinating biomineralization.

### Proteins with high-complexity sequences are unstructured and insoluble

Our previous studies also uncovered the fact that many insoluble proteins with non-degenerative sequences are also highly unstructured. These sequences may escape detection by bioinformatics tools for identifying IUPs as their sequences show no detectable difference from those that adopt well-folded structures. For example, VAPB-3, a splicing variant of VAPB, is both insoluble and unstructured ^84, 85^.

**Figure 3.**
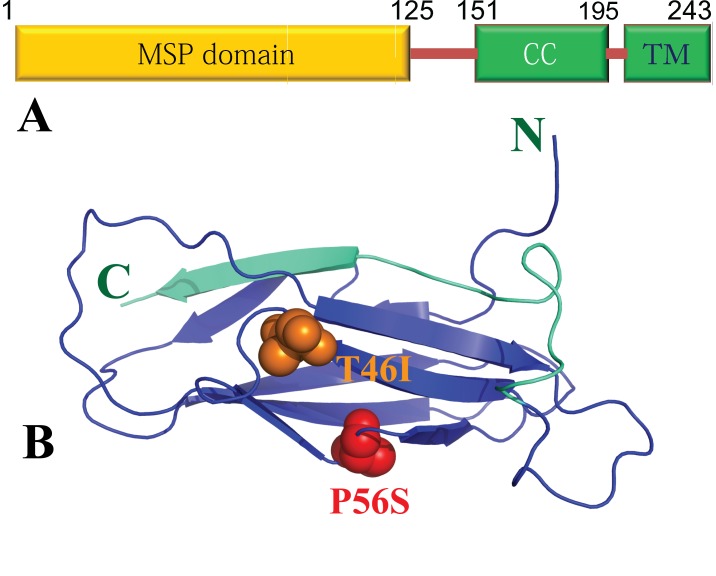
Domain organization and crystal structure of VAPB. ( **A**) 243-residue human VAPB protein is composed of the 125-residue major sperm protein (MSP), coiled coil (CC) and transmembrane (TM) domains. ( **B**) Crystal structure of the 125-residue MSP domain of the human VAPB protein. Two residues, Pro56 and Thr46 are displayed in spheres, whose mutations to Ser and Ile respectively lead to familiar amyotrophic lateral sclerosis. The N-terminal 105 residues (in blue) are identical in both the MSP domain and VAPB-3, a splicing variant of VAPB, while the last 20 residues (in green) of the MSP domain are absent in VAPB-3.

The human VAPB protein is composed of three conserved domains ( Figure 3A), namely an N-terminal 125-residue domain homologous to the major sperm protein (MSP), a central coiled-coil domain, and a C-terminal transmembrane fragment ^67, 79^. Previously we have determined the crystal structure of the human VAPB-MSP domain, which assumes a classic MSP fold with a seven-strand immunoglobulin-like β sandwich ( Figure 3B). Several VAPB splicing variants lacking the transmembrane segment such VAPC and VAPB-3 have been identified to contain truncated MSP domains ^79, 80, 85^. For example, VAPC possesses N-terminal 70 residues that are completely identical to the 125-residue MSP domain of VAPB, and the C-terminal 29 residues unique in VAPC. Interestingly, despite being intrinsically unstructured, VAPC is buffer-soluble and serves as an endogenous inhibitor of HCV infection ^80^.

**Figure 4.**
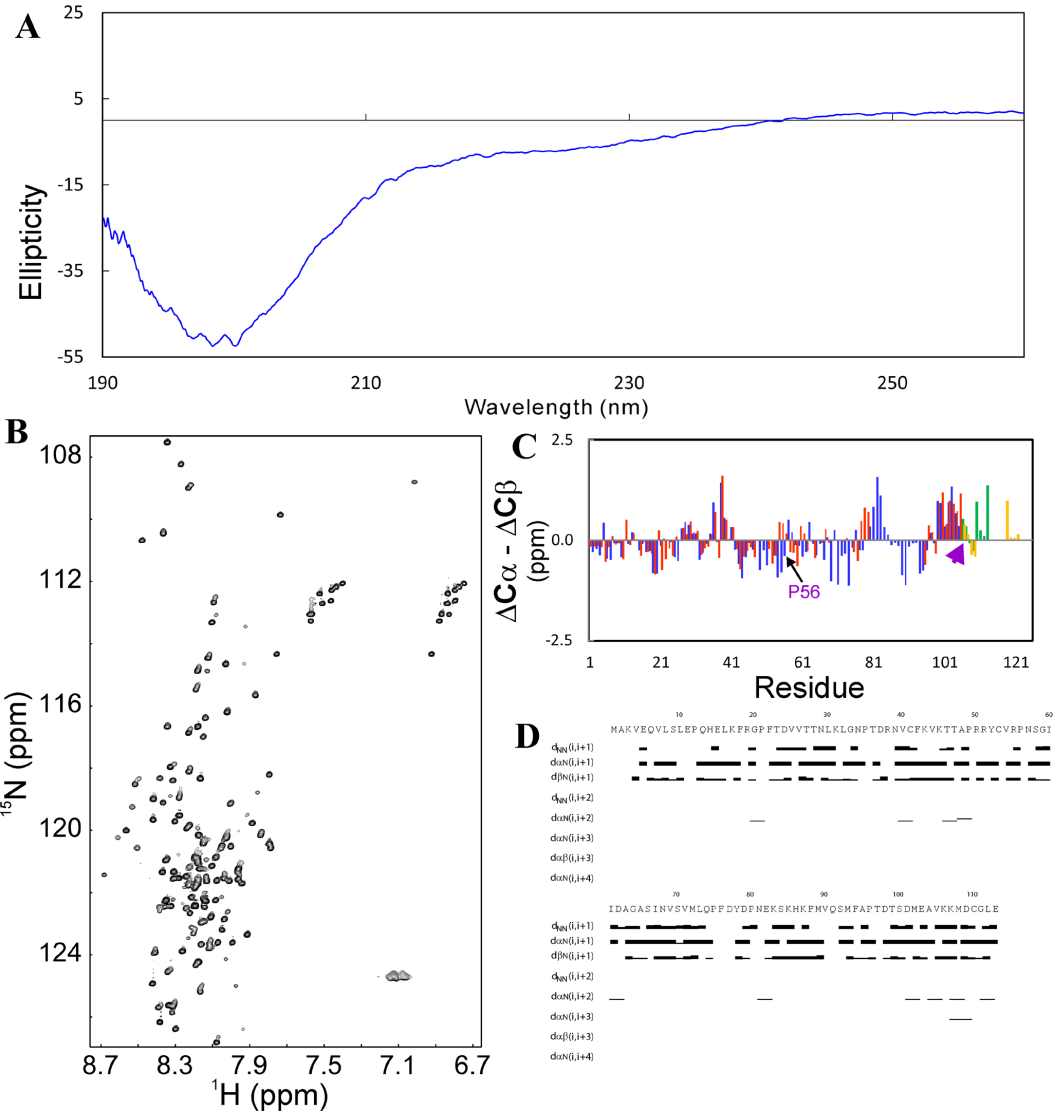
Conformational properties of VAPB-3 solubilized in unsalted water. ( **A**) Far-UV CD spectrum. ( **B**) ^1^H– ^15^N NMR HSQC spectrum acquired at 25ºC. ( **C**) Residue-specific (ΔCα–ΔCβ) values of VAPB-3 and P56S-MSP. For VAPB-3, blue bars are used to indicate (ΔCα–ΔCβ) values for the first 105 residues identical to those of MSP, while green bars for unique residues. For P56S-MSP, red bars are used to indicate (ΔCα–ΔCβ) values for the first 105 residues, while brown bars for the C-terminal 20 residues. ( **D**) NOE connectivity pattern of VAPB-3 defining secondary structures.

In contrast, VAPB-3 is another splicing variant of VAPB, composed of N-terminal 105 residues identical to the MSP domain, plus a short unique C-tail ( Figure 3B). Recently we demonstrated that it was both unrefoldable and insoluble, but again could be solubilized in unsalted water ^84^. CD and NMR characterization indicates that VAPB-3 is predominantly disordered ( Figure 4A–4B). Furthermore, we achieved the sequential assignment of most residues with triple resonance experiments HNCACB and CBCA(CO)NH. Subsequently, we obtained chemical shifts and NOE connectivities by analyzing HSQC-TOCSY and HSQC-NOESY spectra. Very small (ΔCα-ΔCβ) values over the majority of VAPB-3 residues ( Figure 4C) clearly indicate that it is highly unstructured in unsalted water, completely consistent with the CD results. The lacking of stable secondary structures in VAPB-3 is strongly demonstrated by its NOE patterns ( Figure 4D). For most residues, only sequential NOEs could be identified. dαN (i, i + 2), but not dαN (i, i + 3) NOEs were found over several short segments, indicating that the helical conformation weakly populated over these regions is very dynamic. No long-range NOE was detected, as expected for such a predominantly disordered protein without any tight tertiary structure. Based on our CD and NMR analyses, it can be concluded that the truncation in VAPB-3 completely eliminates the intrinsic capacity to fold into the MSP fold, and consequently VAPB-3 no longer has the ability to fold into the MSP fold or any other well-defined structure, thus becoming predominantly disordered in unsalted water. Upon being exposed to salt ions, the exposed hydrophobic side chains will be clustered to result in aggregation through a mechanism I previously proposed ^26^.

### Summary

In this section, I have reviewed the studies on the unrefoldable and insoluble proteins with full-length native sequences. The results reveal that surprisingly a portion of native protein sequences with both low- and high-complexity completely lacks the intrinsic ability to fold into any uniquely defined structures and is also completely insoluble in buffers. While high insolubility of such proteins involved in biomineralization has been recognized to be essential for their functions ^76, 81– 83^, how living systems utilize the insolubility of the proteins represented by ApLLP and VAPB-3 is an important topic to be further explored. Remarkably, the genomes of eukaryotic organisms are anticipated to be abundant in splicing variants, some of which may be similar to VAPB-3, and also have truncation on well-folded domains. As this kind of splicing variants show non-degenerative sequences and high hydrophobicity, they are usually not spotted by the bioinformatics tools for detecting IUPs. Therefore, markedly different from the classic IUPs, VAPB-3 represents a previously undetectable subgroup of IUPs which is characteristic of non-degenerative sequences, with high insolubility and hydrophobicity comparable to that of well-folded proteins. As a consequence, the number of IUPs in eukaryotic genomes may be much larger than currently detected.

Our results also unveil that the mechanism for the misfolding-triggered aggregation shows a fundamental difference from that underlying the aggregation of the proteins represented by ApLLP and VAPB-3. For the 'misfolded proteins', they still retain the intrinsic capacity to fold into well-defined three-dimensional structures, but are misled into aggregation via 'off-pathway' misfolding, triggered by unfavorable conditions such as their over-expression. As such, their aggregation can be significantly recovered with the assistance of cellular chaperone systems. In contrast, the proteins represented by ApLLP and VAPB-3 have no ability to fold into any well-defined structures, due to the low complexity sequences, or truncation of well-folded domains. Therefore, the exposure of hydrophobic side chains in these proteins will unavoidably lead to aggregation upon being exposed to salt ions. Since this category of proteins is naturally occurring, and even in the presence of chaperone systems the aggregation is their evitable destination *in vivo* with ~150 mM ion concentration, here I designate them as 'intrinsically insoluble proteins' (IIPs).

The results also highlight an extreme complexity underlying protein insolubility. Despite having a low hydrophobicity and being unstructured, the full-length ApLLP protein is completely buffer insoluble. In particular, although the nuclear location signaling sequences have no detectable difference in hydrophobicity from the rest of the protein, their deletion renders the truncated ApLLP forms to remain similarly unstructured, but to suddenly become buffer-soluble. This observation imposes a great challenge to precisely predict protein solubility from amino acid sequences by current bioinformatics methods. So why is it so difficult to achieve the prediction? One possibility is that like protein folding, protein aggregation is also highly sequence-dependent. Another scenario has been recently proposed that protein folding/aggregation is a dissipative process of the non-Euclidian manifold in which structural hierarchy builds up by diminishing energy density gradients in the quest for a stationary state determined by surrounding density-in-energy ^4, 5, 86^. The non-Euclidian landscape for protein folding/aggregation is not predetermined. Instead, it is forming and deforming during the folding/aggregation. Hence, the prediction of folding/aggregation is not just technically challenging, but is non-deterministic polynomial time (NP) complete ^87^.

## Solubilizing integral membrane peptide in unsalted water

### Solubilizing an integral membrane peptide in unsalted water

**Figure 5.**
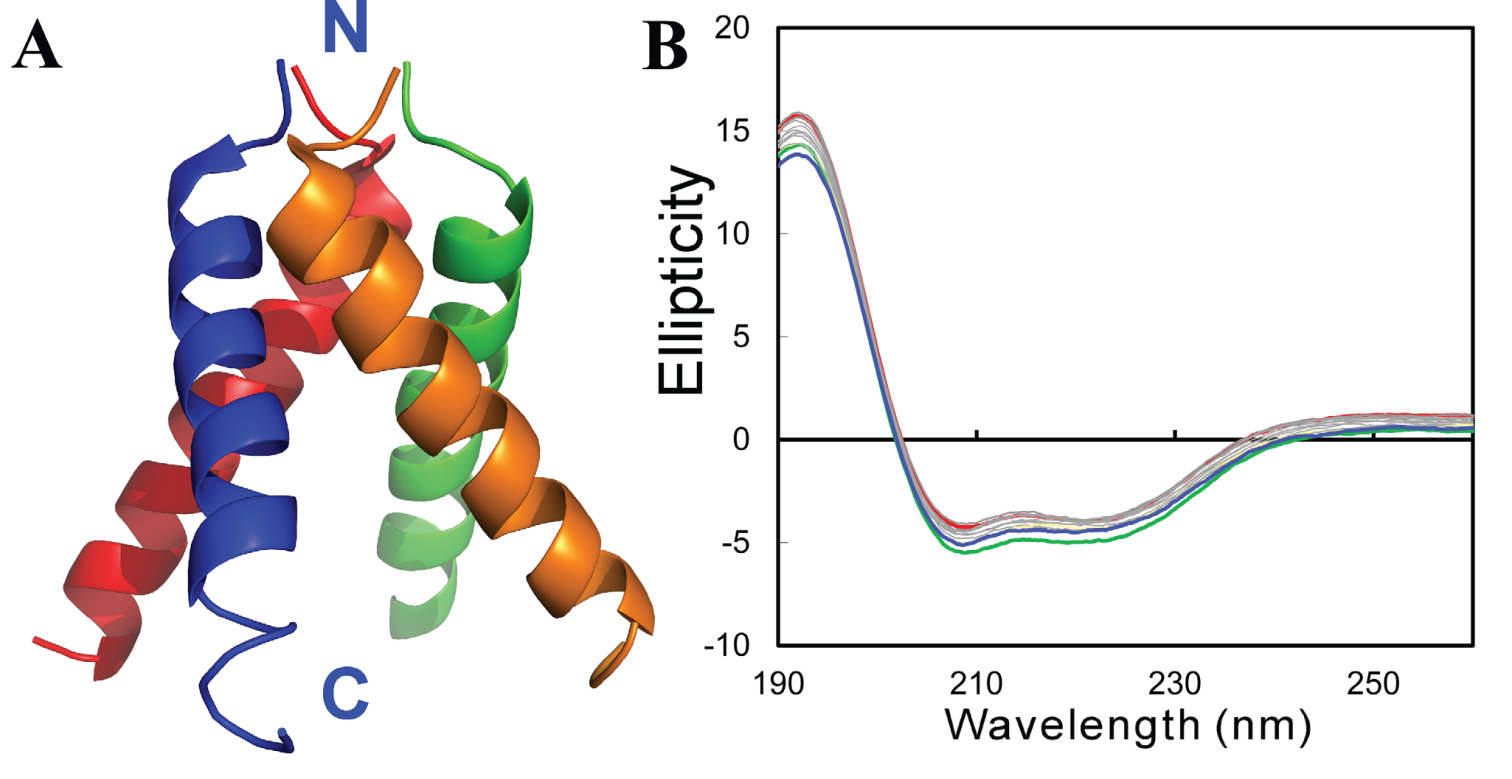
Conformations of the 25-residue integral membrane fragment of the influenza A channel in membrane and unsalted water. ( **A**) Tetrameric structure in a membrane environment with the sequence of SSDPL VVAAS IIGIL HLILW ILDRL. ( **B**) Far-UV CD spectra of the chemically synthetic peptide solubilized in unsalted water (pH 4.0) at a concentration of 100 µM without any lipid molecules, at 20ºC (blue), 95ºC (red) and 20ºC after the thermal unfolding (green).

Even during the preparation of our first two manuscripts on solubilizing insoluble proteins in unsalted water ^21, 68^, I asked myself the question ‘what kind of proteins would be insoluble in unsalted water?’ To assess the upper limit of our discovery, we have attempted, but failed to chemically synthesize and *Escherichia coli* -express peptides containing only bulky hydrophobic residues. Therefore, I decided instead to use the 25-residue M2 transmembrane peptide of the influenza A proton channel, which is one of the most hydrophobic sequences in nature, with a sequence of SSDPL VVAAS IIGIL HLILW ILDRL. Previously, the three-dimensional structures of this peptide have been determined in the presence of lipid molecules by crystallography ^88^, liquid and solid NMR spectroscopy, respectively ^89, 90^. In all these structures, the M2 fragment is a well-formed helix, which is further assembled into a tetramer ( Figure 5A).

To our great surprise, the M2 peptide could again be solubilized in unsalted water to reach at least ~100 μM concentration without any lipid molecules ^91^. Furthermore, in unsalted water, the peptide also forms a high helical conformation which remains almost unchanged during thermal unfolding up to 95ºC, as monitored by CD spectroscopy ( Figure 5B). However, in the absence of lipid molecules, the peptide appeared to be specifically self-organized into a supramolecule complex consisting of a large amount of individual helical peptides as the NMR resonance peaks were too broad to be detected. However, its far-UV CD spectra typical of a helical conformation could not result from non-specific aggregation, as aggregated proteins give rise to far-UV CD spectra similar to that for a β-sheet protein. On the other hand, as expected, the peptide would precipitate immediately upon adding NaCl up to 1 mM.

### Salt ions act as 'dark mediators' for protein aggregation

Now, results by us and other groups have demonstrated that proteins previously thought to be insoluble could indeed be solubilized in unsalted water. In particular, the most hydrophobic integral membrane peptide is also soluble in unsalted water without lipid molecules, but gets aggregated upon the introduction of salt ions. These results together constitute a fact that logically suggests that despite existing in the background, salt ions play at least an equal important role in mediating protein aggregation. Therefore, here I designate salt ions as 'dark mediators' for protein aggregation, analogous to 'dark matter', which controls the global structure of the Universe in the background, but whose nature still remains almost unknown ^92^.

**Figure 6.**
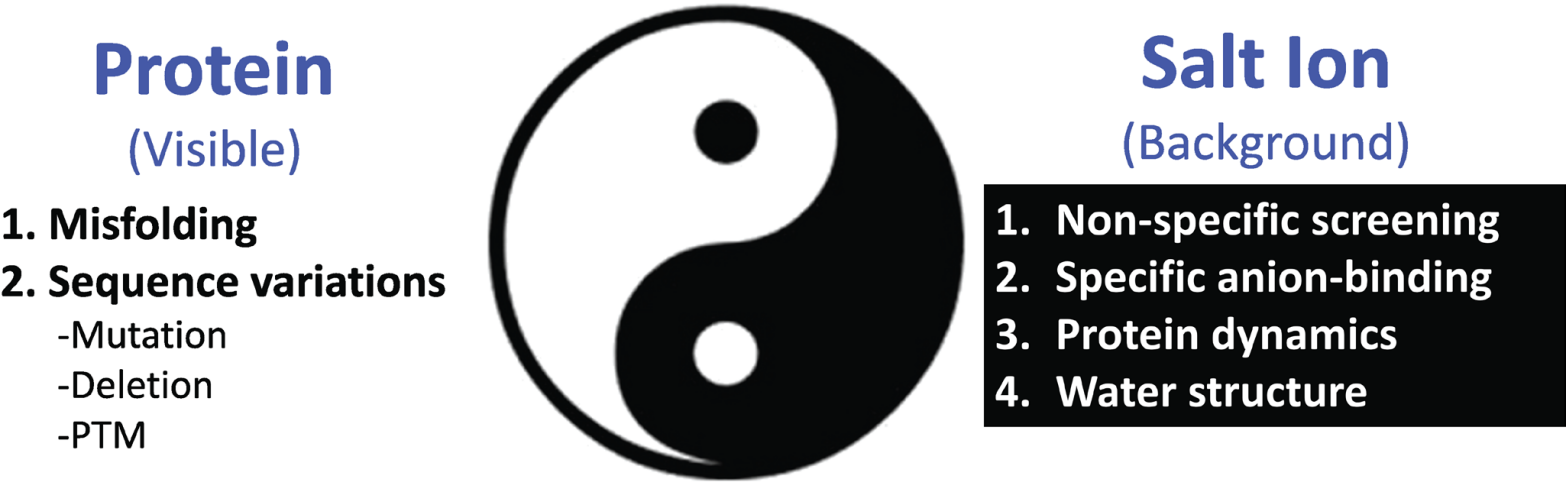
'Yang' and 'yin' of protein aggregation. Factors modulating protein aggregation are symbolized by Taji dagram, which are constituted by those from proteins ('yang') and salt ions existing in the background ('yin'). The 'yang' part has been exhaustively characterized, which includes misfolding and modifications of proteins such as mutation, deletion and post-translational modifications, etc. By contrast, the 'yin' part is only beginning to be recognized, and is involved in the ability of salt ions in imposing non-specific electrostatic screening, specific anion-binding, altering protein dynamics and water structure, etc.

Therefore, in aqueous environments, factors mediating protein aggregation are composed of two key categories, one associated with proteins and another with the salt ions existing in solution. The interplay between them governs protein aggregation and can be symbolized by the Taiji diagram ( Figure 6). While the 'Yang' part from proteins has been clearly recognized and exhaustively assessed, the 'Yin' part from salt ions stays largely elusive in the background, and certainly needs to be explored in the future. In the next sections, I will review our results with both 'Yang' and 'Yin' factors modulating protein aggregation.

## 'Yang' of protein aggregation

Due to the marginal stability and dynamic nature, even wild-type proteins which have the intrinsic capacity to fold into well-defined three-dimensional structures have been extensively demonstrated to 'off-pathway' misfold into aggregates, or amyloid fibers *in vitro* by manipulating solution conditions ^45, 46^. On the other hand, it has been well known that some modifications, especially mutations of certain specific proteins, render them to become unrefoldable and insoluble both *in vivo* and *in vitro*, thus leading to a variety of human disease, in particular neurodegenerative diseases. As a consequence, these modified proteins have been exhaustively characterized by various biophysical methods. However, due to the previous absence of a general method to solubilize these insoluble proteins in the aqueous solution without adding denaturants and detergents, the solution conformations of these unrefoldable and insoluble proteins remained completely unknown. The lack of such knowledge leads to the inability to answer a fundamental question: whether these mutants still possess the intrinsic capacity to fold into well-defined structures, and their aggregation is only due to 'off-pathway' misfolding, or whether they have lost this ability; and consequently aggregate following the mechanism underlying 'intrinsical insoluble proteins'.

With our discovery as a powerful tool, in the past several years we have addressed this question by high-resolution NMR characterizations of several model systems. Below I review two of them. One is an unrefoldable and insoluble SH3 mutant triggered by a single-residue insertion, and another is an unrefoldable and insoluble MSP mutant triggered by one residue P56S mutation, which causes a familiar ALS.

### Insoluble SH3 domain triggered by a single-residue insertion

**Figure 7.**
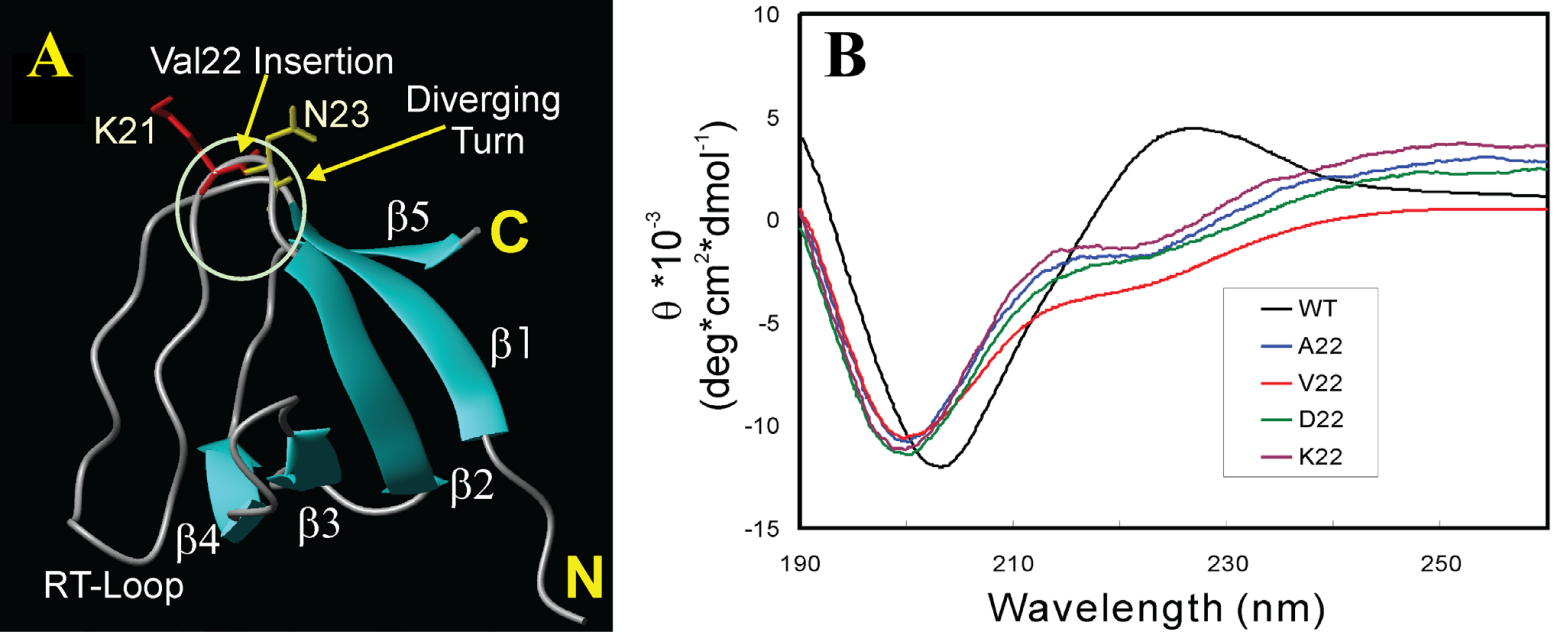
Conformations of wild-type Nck2 SH3 domain and its mutants. ( **A**) NMR structure of the first SH3 domain of the human Nck2 protein (2B86) with the secondary structures and position of the inserted Val residue indicated. ( **B**) Far-UV CD spectra of the wild-type SH3 and its four insertion mutants which were collected at protein concentrations of 20 µM at 25ºC. The wild-type SH3 was dissolved in 5 mM phosphate buffer (pH 6.2) while the insertion mutants were solubilized in unsalted water (pH 4.0).

SH3 modular domains, containing ~60 residues, play a critical role in transmitting, as well as integrating, cellular signals. Structurally, all SH3 domains share a common β-barrel fold comprising five β-stands ( Figure 7A). So far, more than 4,000 SH3 domains have been identified in a variety of organisms ^93– 95^. Very interestingly, we found that the first SH3 domain of the human Nck2 protein, GenBank sequence AAC04831, originally cloned from a tumor tissue, was completely insoluble and not refoldable in all aqueous buffers we tested ^94^. Later on, we found that this sequence had an extra Val insertion at position 22, the tip of the diverging turn linking the RT-loop and the second β-strand ( Figure 7A), thus designated as V22-SH3 ^93^.

**Figure 8.**
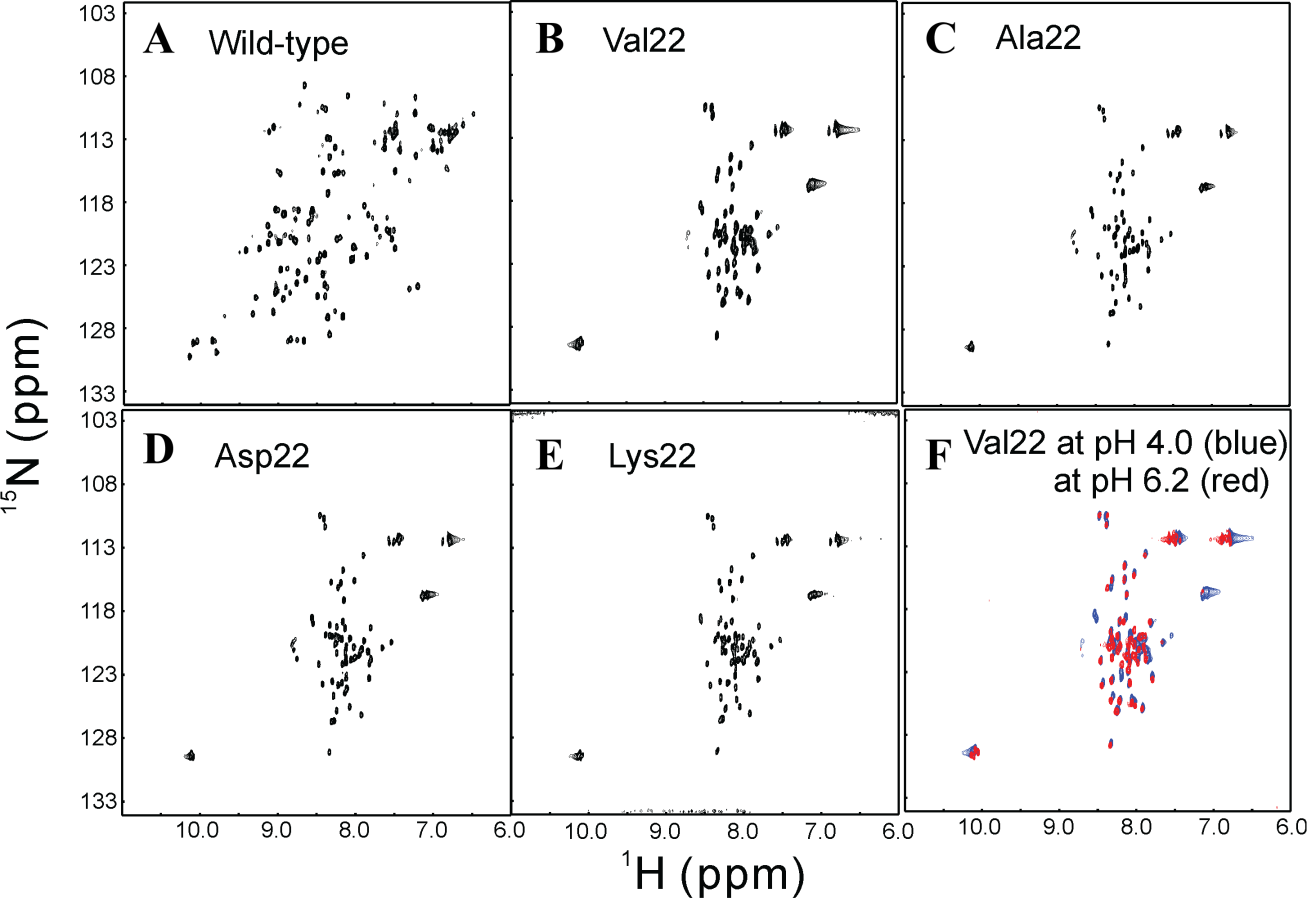
NMR characterization of different forms of the SH3 domain. ^1^H– ^15^N NMR HSQC spectra of the wild-type SH3 and its four insertion mutants at protein concentrations of 100 µM acquired. The wild-type SH3 was dissolved in 5 mM phosphate buffer (pH 6.2) while four insertion mutants were solubilized in unsalted water (pH 4.0). (F). Superimposition of HSQC spectra of V22-SH3 in unsalted water at pH 4.0 (blue) and at pH 6.2 (red).

Nevertheless, with our discovery, we succeeded in characterizing its conformation and backbone dynamics by CD and NMR spectroscopy ^93^. As shown in Figure 7B, far-UV CD spectra clearly indicate that V22-SH3 is highly disordered as compared with the native protein ( Figure 7B). To assess whether the insolubility is a result of the introduction of the bulky Val hydrophobic side chains, we replaced Val with Ala, Asp and Lys residues, respectively. However, the results demonstrated that any of the four insertions at this position would lead to the complete buffer insolubility as well as elimination of the native SH3 fold ( Figure 7B). This notion was further supported by the ^1^H- ^15^N HSQC spectra of these insertion mutants, which have very narrow spectral dispersions at both ^1^H and ^15^N dimensions ( Figure 8). The result thus indicates that the insolubility of V22-SH3 is not due to the introduction of the large Val hydrophobic side chain. It is also worthwhile to point out that no significant difference was identified for the HSQC spectral dispersions of V22-SH3 at pH 4 and 6.2 ( Figure 8F), suggesting that being unstructured is not due to the low pH value.

**Figure 9.**
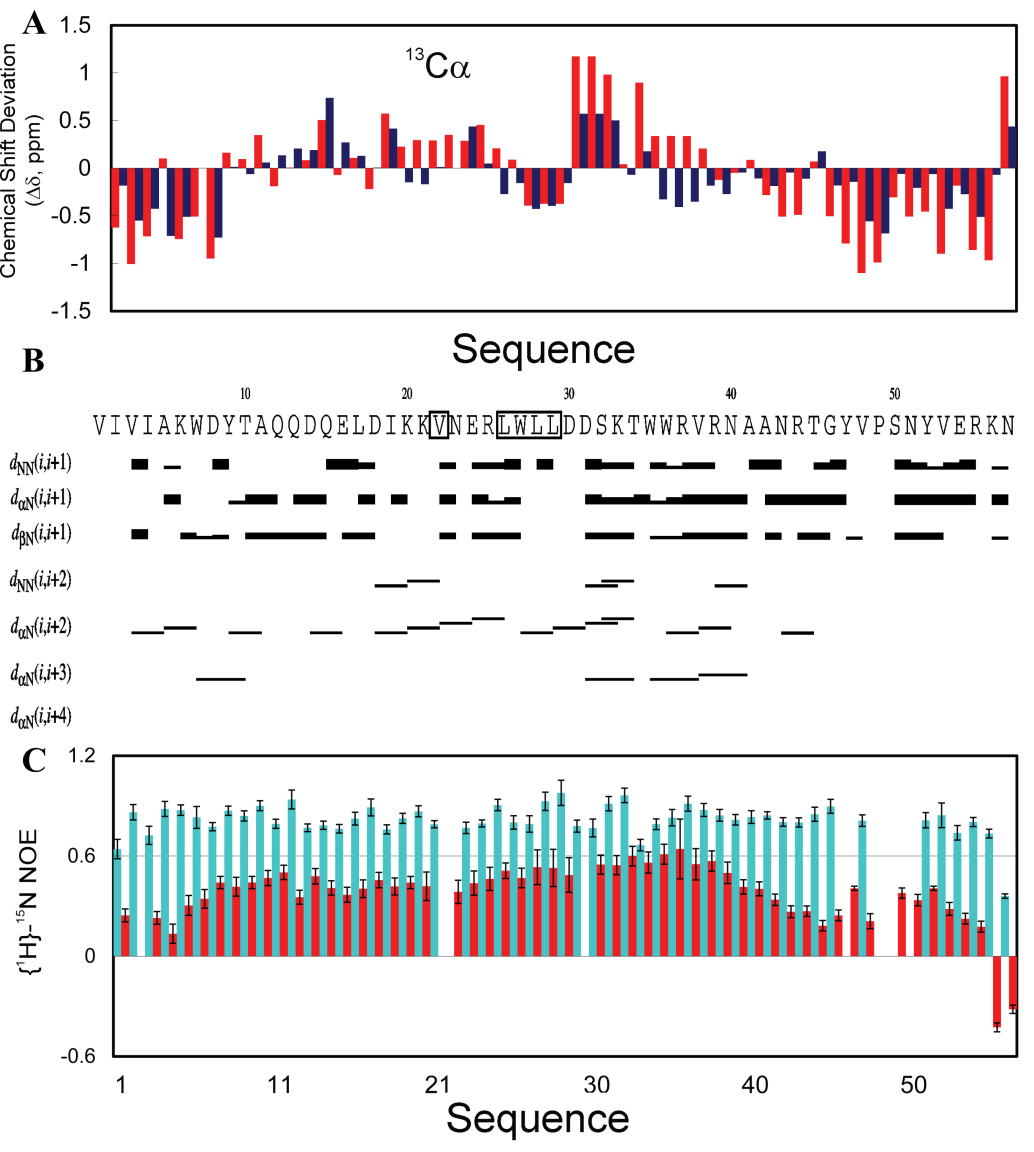
Residue-specific conformations of V22-SH3 in unsalted water. ( **A**) Bar plot of Cα chemical-shift deviations from their random-coil values for V22-SH3 (red), and wild-type in the presence of 8 M urea (blue). ( **B**) Characteristic NOEs defining secondary structures identified for V22-SH3. ( **C**) { ^1^H}- ^15^N steady-state NOE intensities for V22-SH3 (red bars) and wild-type (cyan bars).

We further conducted extensive NMR characterization on conformations and dynamics of the V22-SH3 domain solubilized in salt-free water. As seen in Figure 9A, its Cα chemical shift deviations are already similar to those of the wild-type in the presence of 8 M urea, thus indicating that V22-SH3 becomes significantly disordered ^93^. More specifically, in V22-SH3, the N- (residues 1–6) and C- (residue 47–57) termini are largely unstructured, without any significant secondary structure populated. However, it seems that in V22-SH3, the non-native helical conformation is populated over the secondary region but not the first region, as we have observed on the wild-type SH3 domain at pH 2.0 or 4Ala mutant at pH 6.5 ^95^.

Indeed, in V22-SH3 there exist many non-native αH(i)-NH(i + 2) and αH(i)-NH(i + 3) NOEs over the sequence, in particular over residues 28–42 ( Figure 9B). These NOEs are totally incompatible with the well-formed and rigid β-barrel structure of the SH3 domain in the native condition. As such, the existence of these non-native medium-range NOEs, together with the chemical shift deviations, suggesting that in V22-SH3, the non-native helical conformation is indeed populated over the second region, but not the first region as observed in the wild-type SH3 domain at pH 2.0 and 4Ala mutant at pH 6.5 ^95^. On the other hand, in V22-SH3, there still exist many native-like long-range NOEs ^93^, implying that despite severe disrupted tight packing and populated non-native helical conformations, V22-SH3 may still have a rudiment tertiary topology similar to its native SH3 fold.

We have assessed the backbone dynamics of V22-SH3. As shown in Figure 9C, V22-SH3 has significant reduced hNOE values over the whole sequence if compared with the wild-type SH3 domain at pH 6.5, in particular over the N- and C-termini which are characterized to be highly unstructured by chemical shift deviations. Nevertheless, except for the C-terminal two residues, all V22-SH3 residues still have positive hNOE values, with many >0.4. In particular, hNOE values >0.6 are found for two residues, Trp35 and Trp36, which are located at the central positions of the region that is characterized to own a highly populated helical conformation.

**Figure 10.**
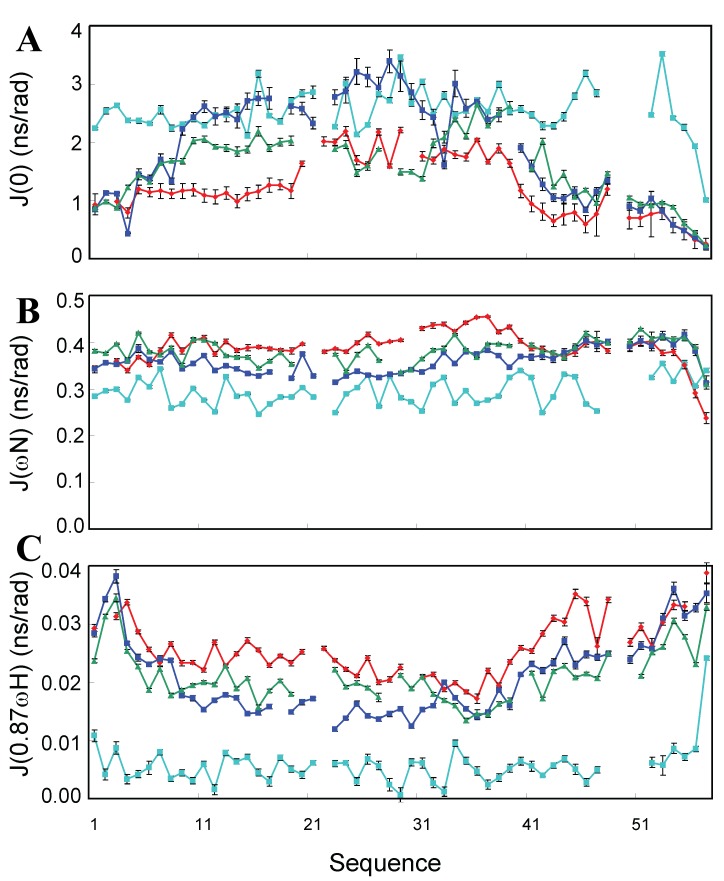
Reduced spectral density function analysis. Spectral densities of V22-SH3 solubilized in unsalted water at pH 4.0 (red); wild-type at pH 6.5 (cyan); wild-type at pH 2.0 (blue) and 4Ala mutant at pH 6.5 (green), calculated from the ^15^N backbone relaxation data measured at 800 MHz. ( **A**) J(0), ( **B**) J(ϖN), and ( **C**) J(0.87ϖH).

Furthermore, we calculated reduced spectral densities at three frequencies from the relaxation data of different forms of this SH3 domain, which reflect relaxation contributions from the motions on different timescales. As seen in Figure 10C, if compared to the wild-type SH3 domain at pH 6.5, V22-SH3, like wild-type SH3 at pH 2.0 and 4Ala mutant, have significantly increased J(0.87ωH) over the whole sequence, indicating that a dramatic increase in the fast motions on the ps-ns timescale. Interestingly, out of three large unfolded SH3 forms, V22-SH3 uniformly has the highest J(0.87ωH) values, suggesting that V22-SH3 has the largest increase of the fast motions. It is particularly interesting to point out that the region with the largest J(0) values are over Glu24-Arg25-Leu26-Trp27-Leu-28-Leu29 ( Figure 10A). This observation implies that this region undergoes slow motions on the µm-ms time scale and/or dynamic aggregation. Strikingly, this region was previously revealed to play a critical role in coordinating the transformation from the non-native helical conformation to native all-β SH3 fold during the folding of this SH3 domain. It is thus likely that in the SH3 domain, there exists an overlap between the interactions responsible for the insolubility and critical for integrating the formation of the native β-barrel fold, as the 4Ala mutant with this region mutated suddenly became soluble in buffer ^95^.

**Figure 11.**
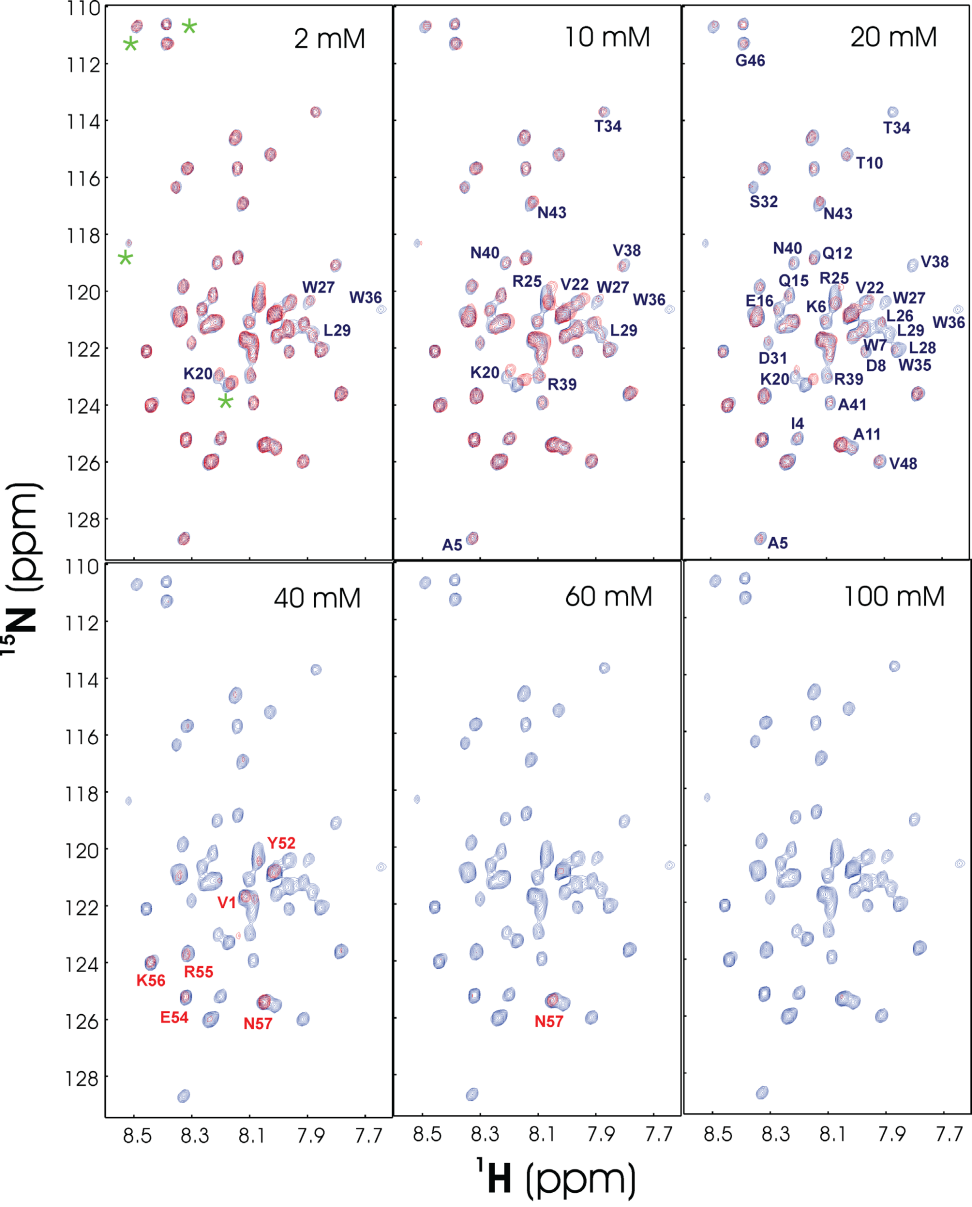
Titrations of V22-SH3 by NaCl salt as monitored by HSQC. Superimposition of the ^1^H– ^15^N NMR HSQC spectra of V22-SH3 at a protein concentration of 50 µM, solubilized in unsalted water (pH 4.0) (blue), and with gradual introduction of NaCl (red) to different concentrations. The HSQC spectra were acquired on an 800 MHz NMR spectrometer at 25ºC. The blue font is used for labeling the residue with its HSQC peak intensity significantly reduced or disappeared, while the red is for the residue with its HSQC peak still observed.

To understand how salt ions affect the conformation and solubility of V22-SH3, we have conducted extensive titrations of NaCl into various V22-SH3 samples solubilized in salt-free water. The results revealed that V22-SH3 has no significantly different conformation in the absence of and in the presence of NaCl, indicating that the failure to form any well-defined structure is an intrinsic feature of V22-SH3. On the other hand, both V22-SH3 and NaCl concentrations are important for its aggregation. If the V22-SH3 concentration is high (>300 µM), addition of NaCl even to 5 mM would result in rapid visible aggregation. As such, we lowered the V22-SH3 concentration down to 50 µM and subsequently collected a series of 1D and 2D HSQC spectra by gradually increasing the NaCl concentrations. As shown in Figure 11, addition of NaCl caused almost no change of the spectral dispersion of V22-SH3, convincingly demonstrating that no fundamental difference exists for its conformations in the absence and presence of salt. However, although no visible aggregate was observed during the experiments, addition of salt even to 2 mM induces the NMR line broadening which leads to the disappearance of some HSQC peaks. This implies that addition of salt, even at a very low concentration, induces dynamic aggregation and/or conformational exchanges on the µs-ms time scale. At a NaCl concentration of 40 mM, most HSQC peaks disappear except for those of several C-terminal residues. When the NaCl concentration reaches 100 mM, all peaks become too broad to be detected. Moreover, after more than 5 hours, the visible aggregates formed in the V22-SH3 sample in the presence of only 5 mM NaCl.

### Insoluble VAPB-MSP domain triggered by an ALS-causing mutation

**Figure 12.**
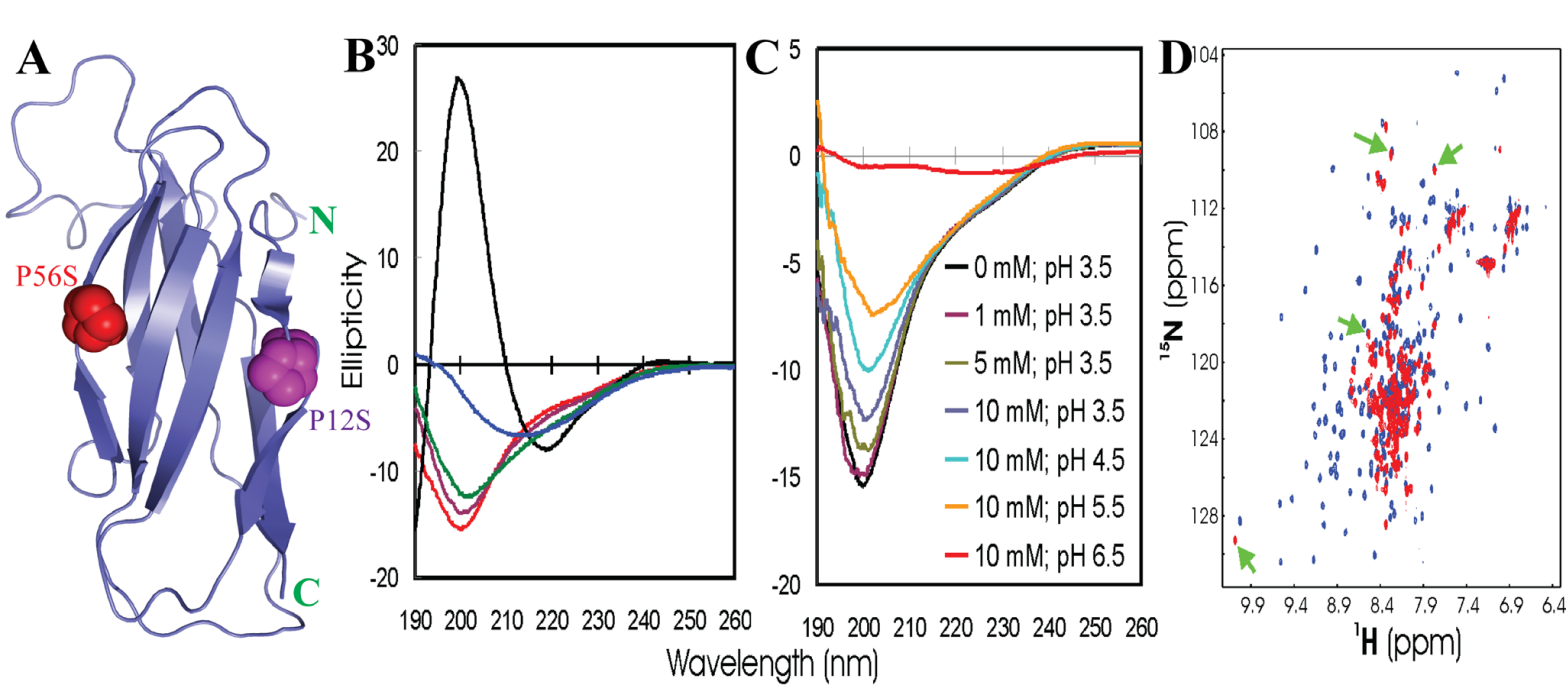
Conformational properties of the wild-type and P56S- MSP domain. ( **A**) Crystal structure of the wild-type VAPB MSP domain, with Pro12 and Pro56 displayed in spheres. The two Pro residues appear to play a key role in maintaining two characteristic S-shaped loops in the MSP domain. ( **B**) Far-UV CD spectra of the wild-type MSP (black) and P56S mutant at pH 3.5 (red), 4.5 (brown), 5.5 (green), and 6.5 (blue). ( **C**) Far-UV CD spectra of the P56S mutant at different pH values and salt concentrations. ( **D**) Superimposition of the HSQC spectra of the wild-type MSP domain (blue) and P56S mutant at pH 3.5 (red).

We also characterized an *in vitro* and *in vivo* insoluble P56S mutant of the VAPB-MSP domain which is a causative mutation for the neurodegenerative disease ALS. This mutant causes a familial ALS, and could not be studied previously due to its high insolubility which is even resistant to solubilization by Triton X-100 ^67, 84^. We first determined the crystal structure of the native VAPB-MSP domain which adopts a seven-strand immunoglobulin-like β-sandwich in which Pro56 and Pro12 adopt the unusual *cis*-peptide bond conformation that appears to be critical in maintaining two characteristic S-shaped loops ( Figure 12A).

Despite showing severe aggregation both *in vivo* and *in vitro*, the P56S mutant protein could again be solubilized at high protein concentrations in unsalted water. Preliminary CD characterization reveals that the P56S mutation eliminates its β-sandwich structure of the wild-type MSP domain, and renders the mutant to be predominantly disordered in water at different pH values ( Figure 12B), and in the presence of NaCl at different concentrations ( Figure 12C). This conclusion is further supported by its narrow HSQC spectral dispersion ( Figure 12D).

**Figure 13.**
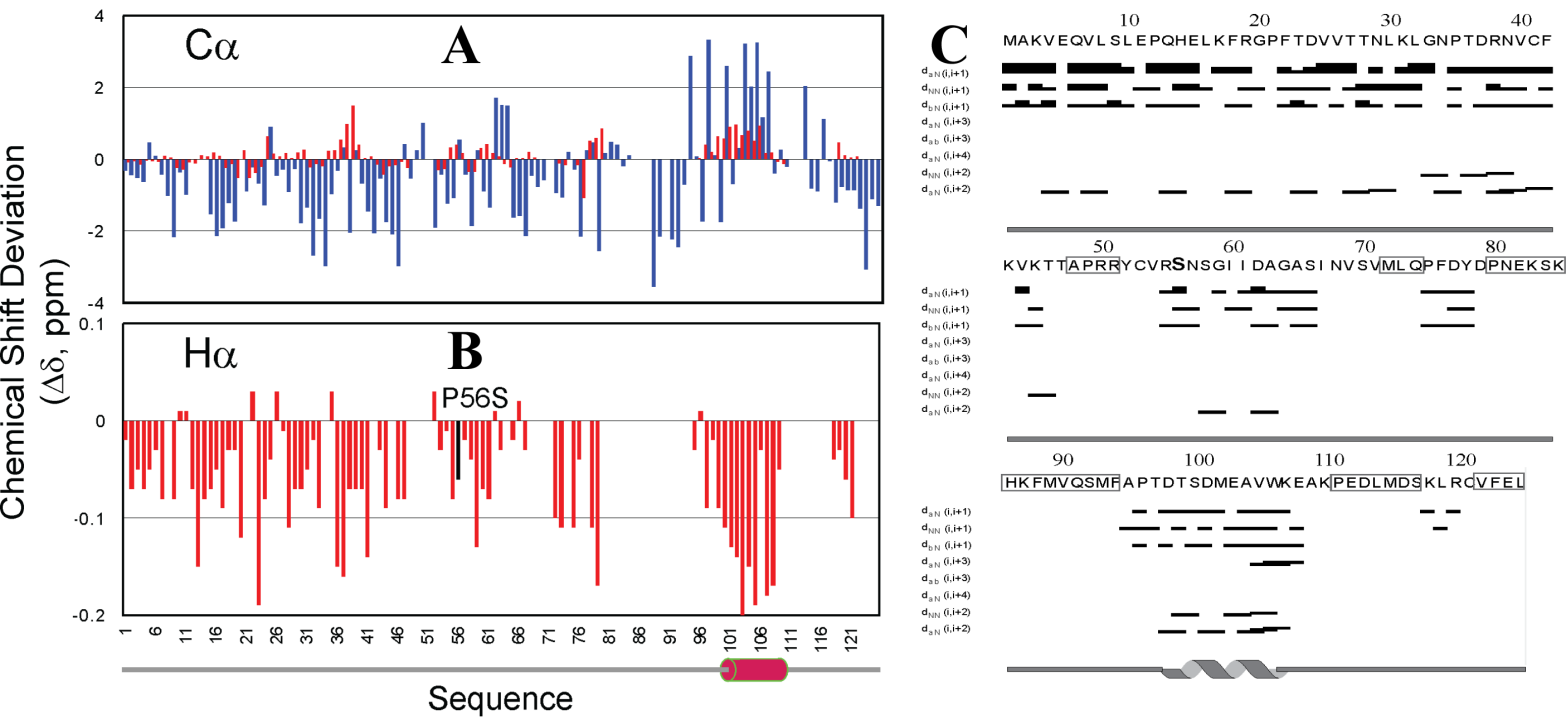
Residue-specific conformations of the P56S-MSP domain in unsalted water. ( **A**) Bar plot of chemical shift deviations (Δδ = δobs - δcoil) of Cα atoms from their random-coil values for the wild-type (blue) and P56S (red). ( **B**) Chemical shift deviations of Hα atoms for P56S. ( **C**) Characteristic NOEs defining secondary structure identified for the P56S mutant.

We have performed detailed NMR characterization of both wild-type and P56S MSP domains by acquiring a large set of three-dimensional heteronuclear NMR spectra at a 500 µM protein concentration. As shown in Figure 13A, the wild-type MSP domain has very large Cα chemical shift deviations typical of a fully folded protein. By contrast, the P56S mutant has dramatically reduced deviations characteristic of an unfolded protein. Very surprisingly, it appears that in Pro56Ser, the native β-sheet secondary structure is totally eliminated. Instead, based on the negative Hα chemical shift deviations ( Figure 13B), it appears that the non-native helical conformation is weakly populated over the sequence, in particular over residues Thr97-Glu108. In the wild-type MSP structure, a helix is also formed, but it is much shorter, only over Ala104-Glu108. Further analysis of the NOE connectivity pattern ( Figure 13C) indicates that except for the missing residues, sequential dNN(i, i + 1) and medium-range dRN(i, i + 2) NOEs could be observed over the majority of the sequence, suggesting that the non-helical conformation is indeed populated to some degree. However, dNN(i, i + 2) NOEs could be found only over two segments (Gly33-Thr46 and Thr97-Glu108), while only two dαN(i, i + 3) NOEs could be identified between Ala104 and Lys107, and between Val105 and Glu108. As such, the non-native helical conformation is only dynamically populated in the P56S mutant.

**Figure 14.**
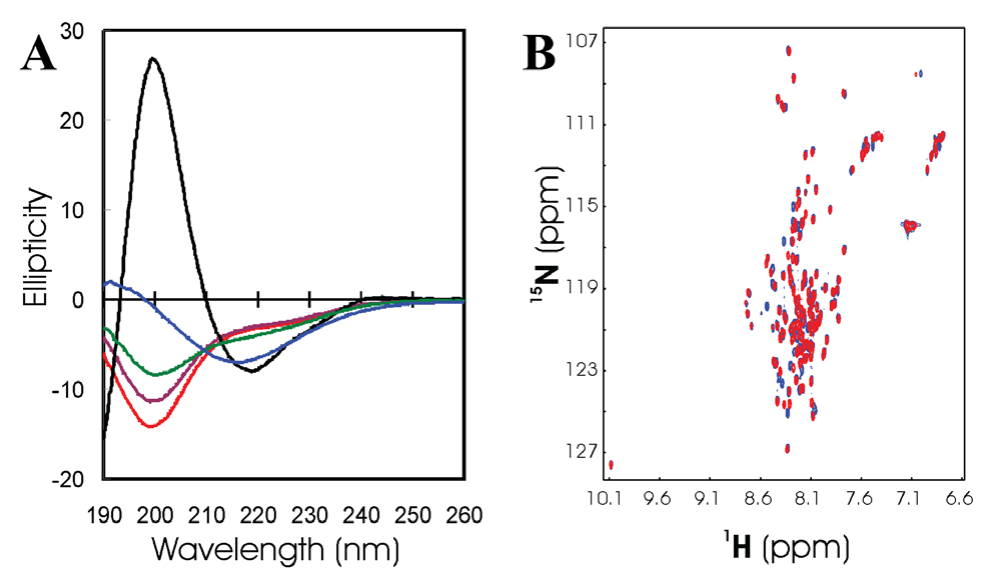
Conformational properties of the P12S-MSP domain. ( **A**) Far-UV CD spectra of the wild-type MSP (black) and P12S mutant in unsalted water at pH 3.5 (red), 4.5 (brown), 5.5 (green), and 6.5 (blue). ( **B**) Superimposition of HSQC spectra of P56S (blue) and P12S mutants in unsalted water at pH 3.5 (red).

Interestingly, as seen in Figure 12A, two characteristic S-shaped loops constrained by P12 and P56 respectively, are present in the MSP domain. Thus we have explored whether Pro12 also plays a similar role to P56 in maintaining the MSP fold. The obtained results showed that indeed the P12S mutant also became unrefoldable and insoluble, but again could be dissolved in unsalted water. CD studies indicate that P12S is highly unstructured at different pH values ( Figure 14A). This conclusion is further supported by its HSQC spectra at different pH values. In particular, most HSQC peaks of the P12S mutant can be superimposed with those of the P56S mutant ( Figure 14B), strongly suggesting that the P12S and P56S mutants share similar conformational properties over the majority of the molecules.

### Summary

We have characterized conformations of unrefoldable and insoluble proteins resulting from one-residue insertion/mutation on the well-structured folds such as SH3 and MSP shared by a large number of sequences. The results uncover a surprising fact that such one-residue variations, which also occur naturally, are sufficient to completely eliminate the intrinsic capacity of the wild-type sequences to fold into the native folds, thus rendering the mutants to become 'intrinsically insoluble proteins'. Therefore, in addition to the low complex sequences and truncation of well-folded domains as exemplified by ApLLP and VAPB-3, the mutation and insertion of well-folded domains represents another mechanism underlying the emergence of 'intrinsically insoluble proteins' in genomes. As eukaryotic genomes contain a large number of random mutations, it is thus reasonable to expect that part of these mutations may lead to 'intrinsically insoluble proteins'.

The results also strongly suggest that there indeed exists a small set of critical residues which make a dominant contribution to the formation/maintenance of the native structures ^17^. Intriguingly, as shown in the SH3 domain, the interactions critical for coordinating the formation of the SH3 fold have an overlap with those responsible for its aggregation. This might represent a general phenomenon which implies that even for proteins adopting well-folded structures, nature may not or may be unable to optimize their sequences to completely avoid aggregation.

## 'Yin' of protein aggregation

### No fundamental difference for conformations in unsalted and salted water

So far, my lab has encountered ~60 unrefoldable and insoluble proteins; and we found that they were all soluble in unsalted water but lacking in well-defined structures. The same results have also been reported by other groups on insoluble proteins including those involved in biomineralization. On the other hand, when I presented speeches on our discovery in conferences, one always-asked question is whether being unstructured for such insoluble proteins solubilized in unsalted water is due to the absence of salt ions. Although previously we have addressed this question by titrating unrefoldable and insoluble proteins solubilized in unsalted water with NaCl, and demonstrated that the addition of NaCl triggered no formation of well-folded structures, NaCl, however, is generally considered to be neutral in the Hofmeister series and therefore it is of fundamental interest to evaluate the effects of other salts.

**Figure 15.**
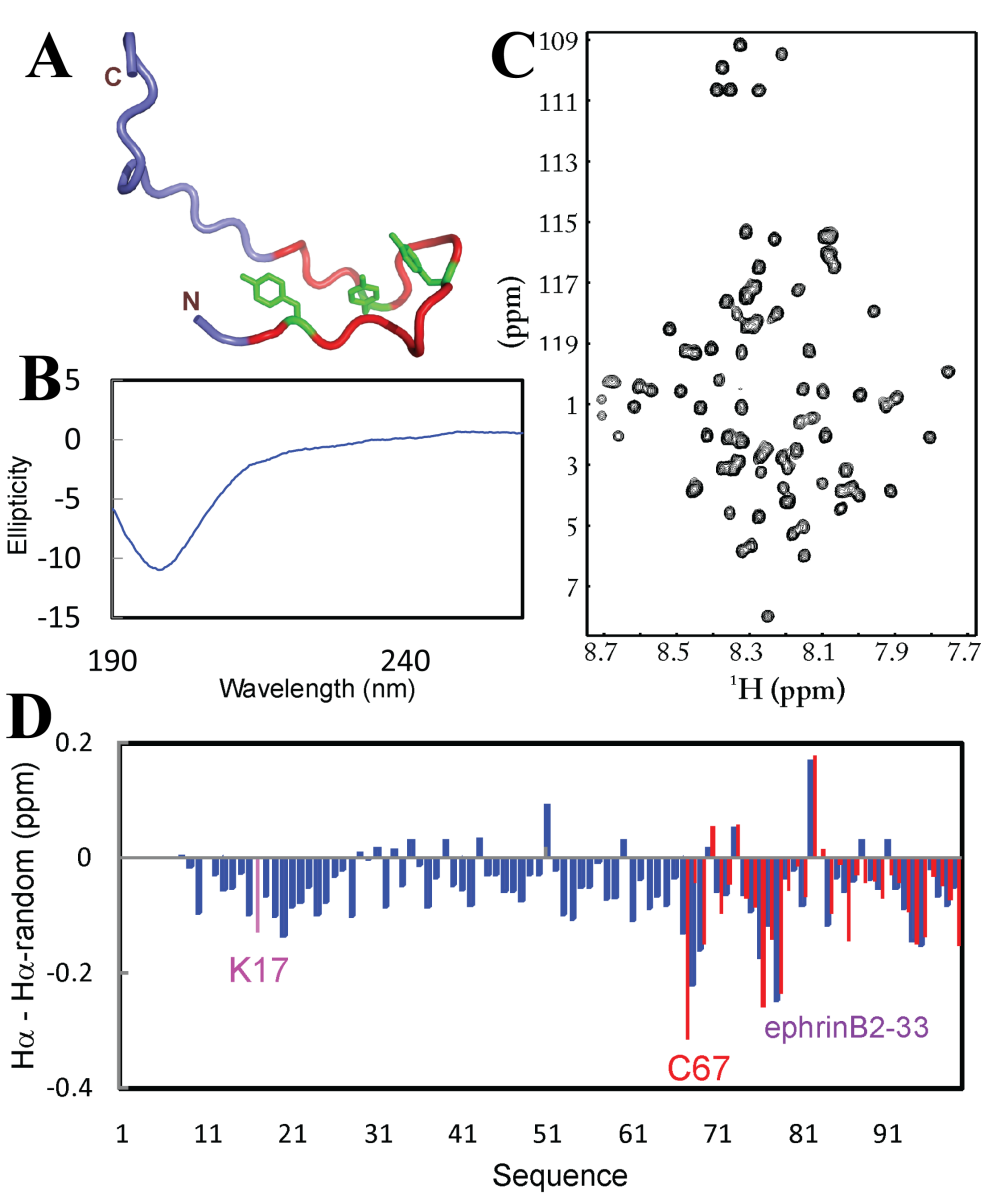
Conformations of the entire cytoplasmic domain of ephrin-B2 (83 residues) solubilized in unsalted water. ( **A**) Three-dimensional structure of the last 33 residues designated as ephrinB-33 that we previously determined by NMR spectroscopy. ( **B**) Far-UV CD spectrum of the entire ephrin-B2 cytoplasmic domain at a protein concentration of 20 µM (pH 4.0) at 25ºC. ( **C**) ^1^H– ^15^N NMR HSQC spectrum at a protein concentration of 300 µM (pH 4.0). ( **D**) Hα conformational shifts of the entire ephrin-B2 cytoplasmic domain with a 16-residue His-tag (blue bars) and of the isolated last 33 residues ephrinB-33 (red bars). The first 7 residues of the His-tag could not be assigned due to missing side-chain resonances. Lys17 is the starting residue of the entire cytoplasmic domain while Cys67 is the starting residue of ephrinB-33.

We thus extended our previous investigations by titrating the 83-residue cytoplasmic domain of ephrin-B2 with 14 salts whose 8 anions are located in the middle, on the left and right sides of the Hofmeister series ^96^. Previously the entire domain was found to be insoluble and consequently its structure remains unstudied ^97^. However, by a truncation approach, the last 33 residue functional subdomain was found to be soluble even in 50 mM sodium phosphate buffer and consequently its NMR structure was determined to assume a well-packed hairpin followed by largely unstructured C-terminal 11 residues ( Figure 15A). Interestingly, the phosphorylation of three Tyr residues on the β-hairpin would disrupt the structure and lead to the binding to the downstream Nck2-SH2 domain for signal transduction ^98^. Therefore, the last 33 residues can serve as an internal reference to report its conformations in buffer and unsalted water.

The full-length domain was indeed buffer-insoluble but again could be solubilized in unsalted water. Preliminary characterizations by far-UV CD ( Figure 15B) and NMR HSQC spectroscopy ( Figure 15C) indicate that the entire domain is largely unstructured without any tight tertiary packing. On the other hand, we have achieved the sequential assignment of the whole domain; and the Hα conformational shifts support the conclusion that the entire domain is largely disordered ( Figure 15D). Most strikingly, the last 33 residues of the entire domain in unsalted water have Hα chemical shifts almost identical to the isolated 33-residue subdomain in 50 mM sodium phosphate buffer. This clearly indicates that the last 33 residues adopt almost the same conformation in unsalted and buffer water.

**Figure 16.**
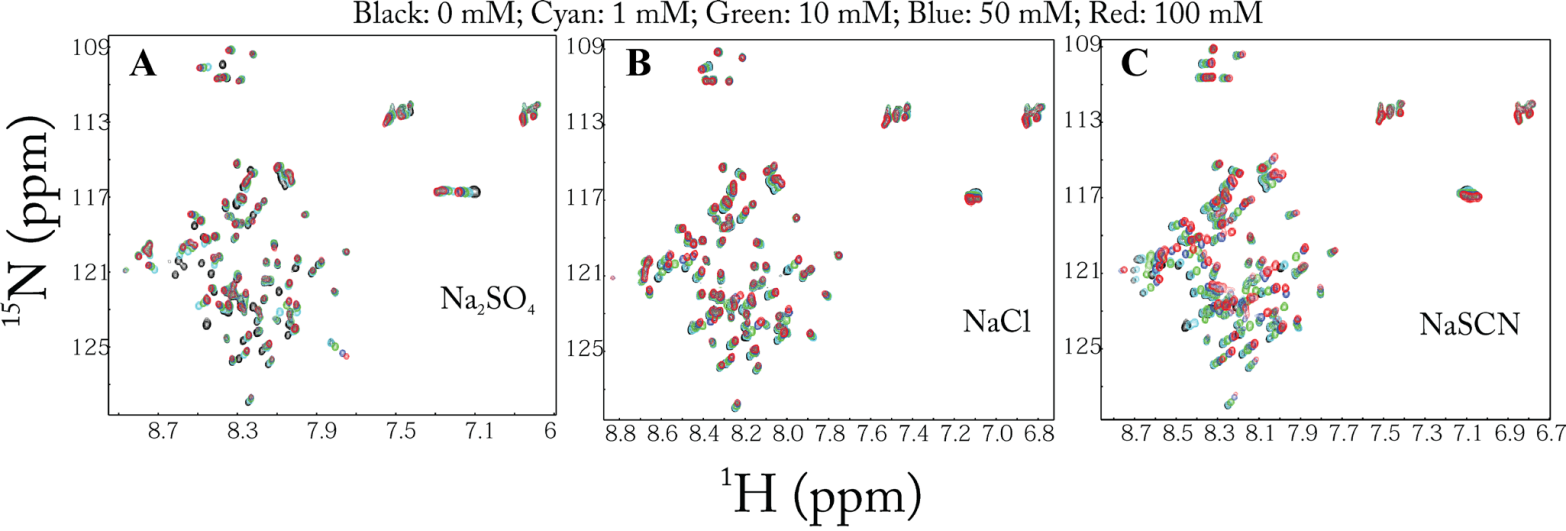
Anion-binding as monitored by NMR. Superimposition of two-dimensional ^1^H– ^15^N NMR HSQC spectra of the entire cytoplasmic domain of ephrin-B2 at a protein concentration of 300 μM (pH 4.0) at 25ºC, in the absence and in the presence of Na _2_SO _4_, NaCl and NaSCN at varying salt concentrations.

We subsequently monitored conformational changes by HSQC spectroscopy upon titrating the domain with 14 salts: namely Na _2_SO _4_, NaF, NaSCN, Na _2_HPO _4_, NaCl, NaBr, NaNO _3_, NaI, MgCl _2_, KCl, CaCl _2_, guanidinium chloride (GdmCl), LiF, and KCl. As exemplified by the HSQC spectra with Na _2_SO _4_ located on the left, NaCl in the middle, and NaSCN on right sides of the Hofmeister series ( Figure 16), gradual introduction of three salts up to 100 mM results in no significant change of the HSQC spectral dispersions, but only slight shifts of HSQC peaks. The results clearly demonstrate that no significant conformational change occurs upon adding 14 salts up to 100 mM. Therefore, being unstructured for unrefoldable and insoluble proteins solubilized in unsalted water is not a result of the absence of salt ions, but reflects their absence of the intrinsic capacity to form any well-folded structures.

### Anion-specific binding with high affinity and selectivity

**Figure 17.**
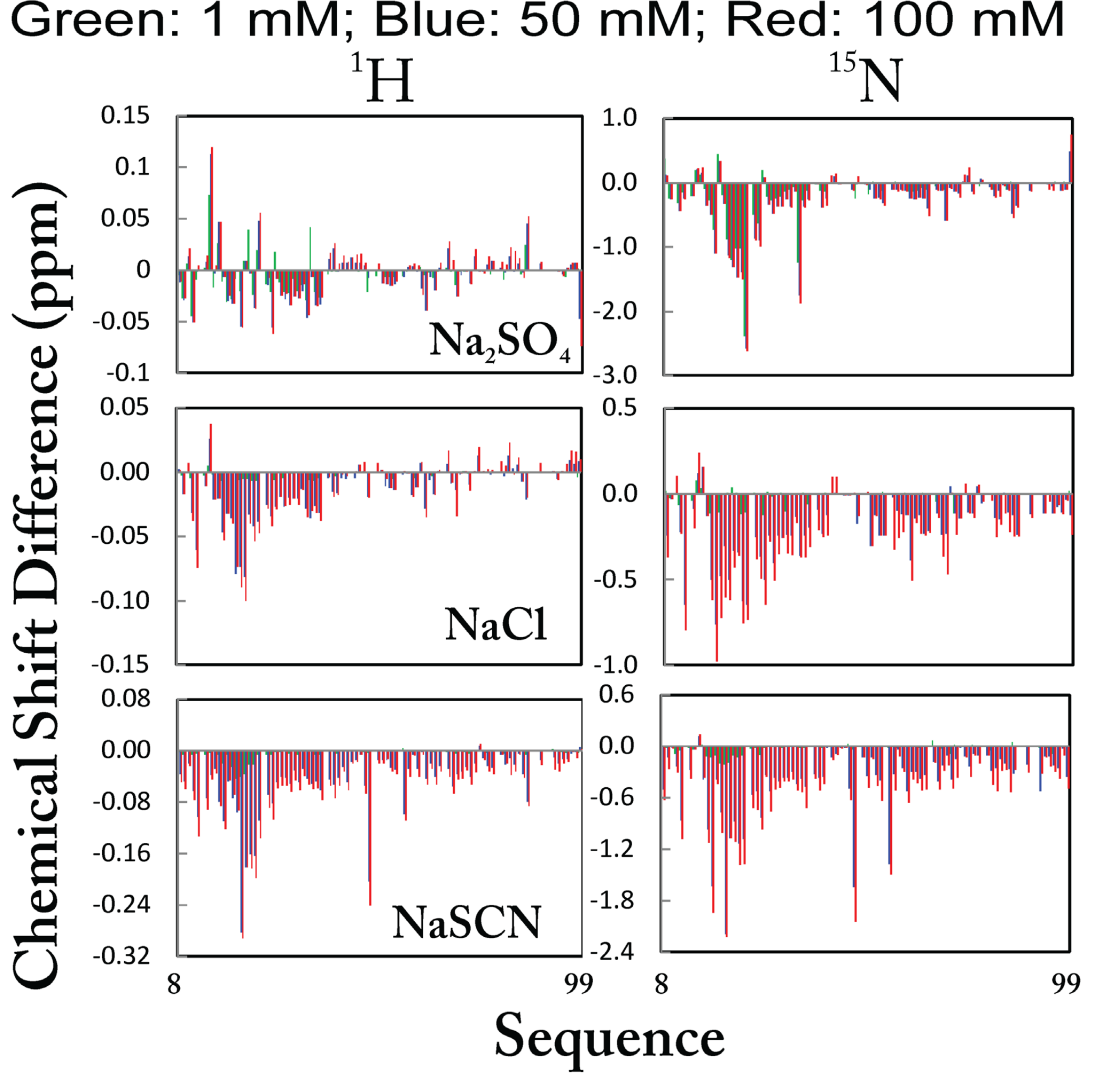
Specific anion-binding as revealed by NMR. Chemical shift difference (CSD) of the amide proton ( ^1^H) and nitrogen ( ^15^N) for residues of the entire ephrin-B2 cytoplasmic domain plus His-tag induced by the addition of Na _2_SO _4_, NaCl and NaSCN at three different concentrations.

To our surprise, however, the salt effects on shifting of HSQC peaks are not uniform over the whole protein, as represented by Figure 16. By superimposing the HSQC spectra at different salt concentrations, we mapped out the residue-specific changes of ^1^H and ^15^N chemical shifts upon salt titrations, as represented by the results with three salts ( Figure 17). Although upon addition of salts, most of the residues with HSQC peaks shifts are located over the N-terminal half of the protein, the patterns of the chemical shift changes are very diverse for salts whose anions are located on the left side of the Hofmeister series, while they are largely uniform for those anions that are on the right side of the series including NaCl and NaSCN. To determine the relative contribution of cations and anions to the HSQC peak shifts induced by salts, we monitored the shifts by titrating chloride salts with different cations, including MgCl _2_, KCl, CaCl _2_, and GdmCl. The different chloride salts caused very similar patterns of HSQC peak shifts, suggesting that the observed effects are mostly due to the chloride anion ^96^. These results suggest that the HSQC peak shifts observed here are mostly triggered by the asymmetric binding of different anions to the protein residues.

**Figure 18.**
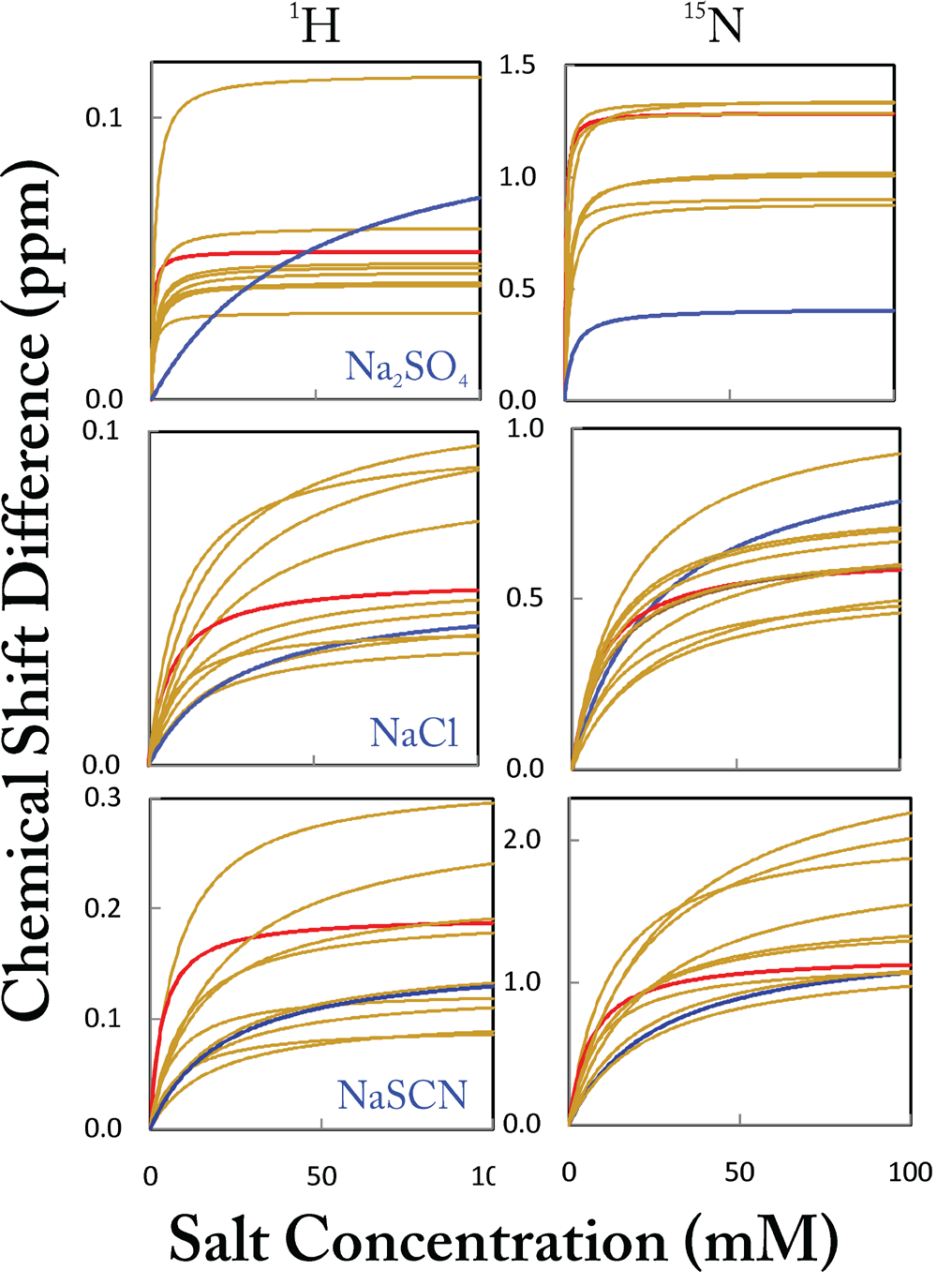
Apparent dissociation constants for specific anion-binding. The fitting curves for ^1^H and ^15^N chemical shift changes using the one binding site model for significant shifted residues of the entire ephrin-B2 cytoplasmic domain, which are induced by gradually adding Na _2_SO _4_, NaCl and NaSCN. The blue and red lines are used to indicate the curves associated with the highest and lowest Kd values, respectively.

We then plotted the chemical shift changes induced by ions as a function of salt concentration for the residues that demonstrate significant peak shifts, and very unexpectedly, all curves appear to be saturable ( Figure 18). This implies that these anions all have specific binding to the protein. Using the one binding site model, we separately fitted these titrations based on ^1^H and ^15^N chemical shifts. To our surprise again, the majority of the apparent Kd values are found to be less than 50 mM, thus indicating that all eight anions bind to the ephrin-B2 cytoplasmic domain with high affinity. Of particular significance, Na _2_SO _4_ is found to be the strongest binder to most residues, with an average apparent Kd of only 1 mM ^96^.

### Specific anion-binding to a well-folded protein

Our study on the unstructured ephrin-B2 cytoplasmic domain ^96^ suggests that the protein-anion interaction is mostly modulated by anion type, protein conformation and electrostatic property. Therefore, a high-resolution study of the effects of different salts on a well-folded protein is crucial. We thus investigated interactions of three physiologically relevant salts with the well-folded WW4 domain ^99^. Previously we have determined its NMR structure and binding with a Nogo-A peptide ^100^. By use of the same NMR HSQC titrations, we monitored its binding to three salts (Na _2_SO _4_, NaCl and NaSCN) with salt concentrations up to 200 mM (800 protein molar equivalents), under three solution conditions: 1) in unsalted water at pH 6.4; 2) in 20 mM phosphate buffer at pH 6.4; and 3) in water at pH 4.0.

**Figure 19.**
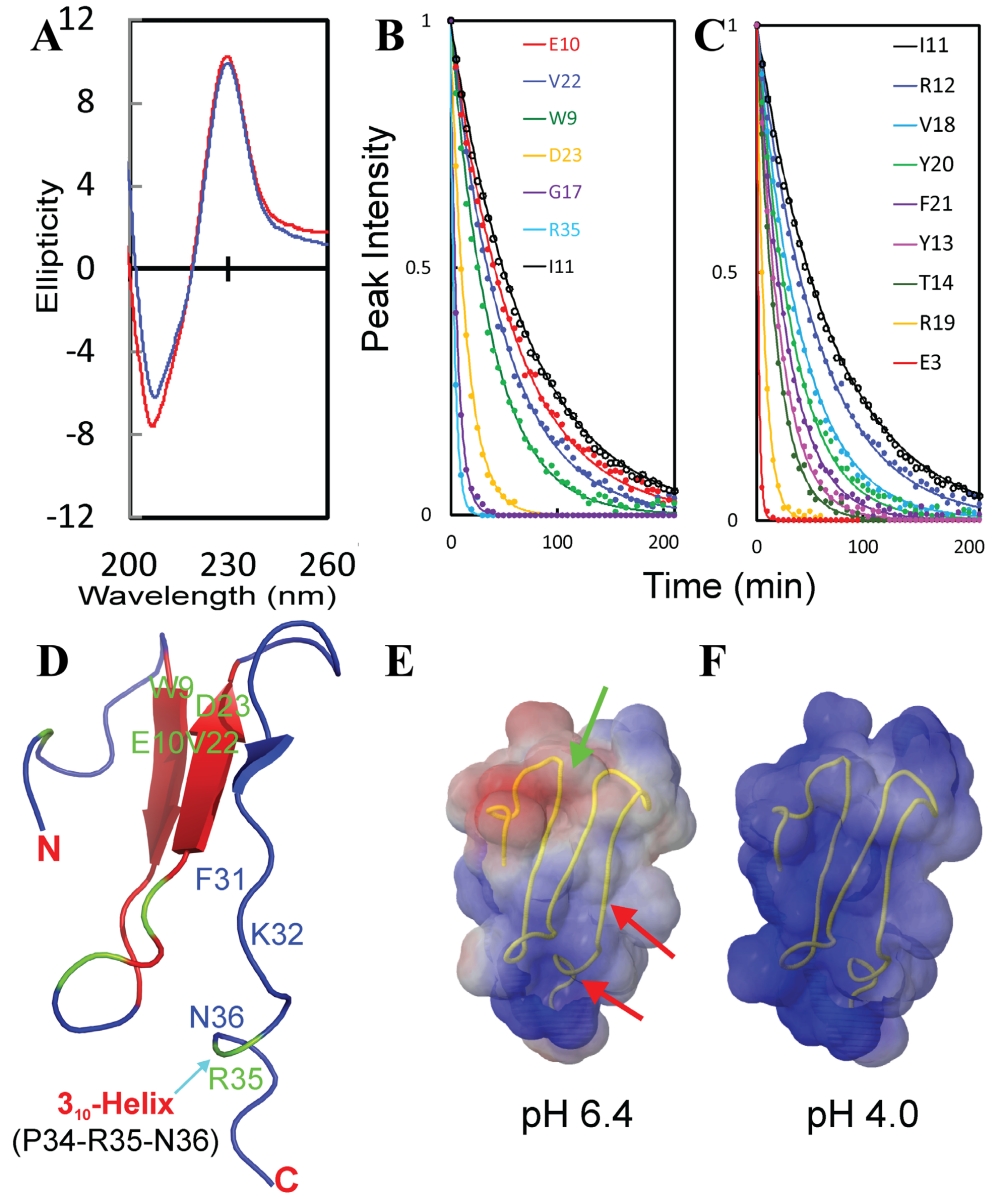
Conformations, accessibility and electrostatic potential surfaces of the well-folded WW4 domain. ( **A**) Far UV CD spectra of the WW4 domain (25 μM) in unsalted water at pH 6.4 (blue) and 4.0 (red). ( **B**)–( **C**) Experimental (dots) and fitted (lines) values are shown for HSQC peak intensities in NMR H/D exchange experiments for WW4 residues whose peaks are significantly ( **B**) and not significantly ( **B**) perturbed by salts. ( **D**) WW4 structure colored with H/D exchange rates (Kex): blue for residues with HSQC peaks disappeared in 2 min after the lyophilized sample was dissolved in D _2_O; green for residues with Kex >5 h ^-1^ and red for residues with Kex <5 h ^-1^. ( **E**)–( **F**) The electrostatic potential of WW4 at pH 6.4 and 4.0 respectively, which is calculated with APBS and visualized at the level of the accessible surface of the protein, with blue and red corresponding to positive and negative potential values respectively.

**Figure 20.**
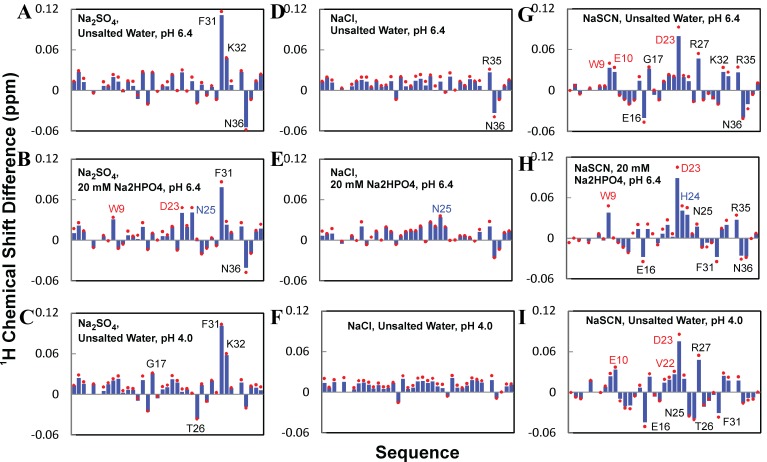
Anion binding to WW4 as monitored by NMR. Residue-specific chemical shift differences (CSD) of amide protons of the WW4 domain triggered by titration of Na _2_SO _4_, NaCl and NaSCN. ( **A**)–( **C**) Na _2_SO _4_; ( **D**)–( **F**) NaCl; and ( **G**)–( **I**) NaSCN titrations of WW4 in unsalted water at pH 6.4; in 20 mM phosphate buffer at pH 6.4, and in water at pH 4.0 respectively. Residues with significant ^1^H chemical shift changes (>0.03 ppm) are labeled: red for residues with amide proton H/D exchange rate (Kex) <5 h ^-1^ and blue for residues with significant changes only in the presence of 20 mM sodium phosphate buffer (pH 6.4).

As seen in Figure 19A, WW4 has a far-UV CD spectrum characteristic of a β-turn rich protein, and no significant difference is found for its spectra in water at pH 6.4 and 4.0, demonstrating that WW4 has very similar structures at two pH values. Furthermore, we determined the exposure degree of the WW4 amide protons to solvent by NMR H/D exchange experiments ( Figure 19B–D), as well as calculated its electrostatic potential surfaces at pH 6.4 ( Figure 19E) and 4.0 ( Figure 19F). Under three solution conditions, we acquired a series of HSQC spectra of WW4 with progressive addition of Na _2_SO _4_, NaCl and NaSCN. The results showed that addition of three salts up to 200 mM leads to no dramatic change of HSQC spectral dispersions, suggesting no significant alternation of tertiary packing. Nevertheless, addition of three salts does induce shifts of distinctive sets of HSQC peaks. Specifically, in water at pH 6.4, NaCl induces significant shifts (>0.03 ppm) for only two residues (Arg35 and Asn36) ( Figure 20D); Na _2_SO _4_ for three (Phe31, Lys32 and Asn36) ( Figure 20A) and NaSCN for nine (Trp9, Glu10, Glu16, Gly17, Asp23, Arg27, Lys32, Arg35 and Asn36) ( Figure 20G). Markedly, the overall shift patterns induced by each salt are highly similar in unsalted water at pH 6.4 and 4.0.

We fitted all titration tracings with ^1^H chemical shift differences >0.03 ppm to obtain the apparent dissociation constants (Kd) based the one binding site model ( Table 1). Intriguingly, although Na _2_SO _4_ perturbs far fewer numbers of residues than NaSCN, it has the strongest binding affinity, with average Kd values of 32.0, 15.7 and 86.3 mM respectively for backbone amide protons under three conditions. NaCl and NaSCN have similar affinity but lower than Na _2_SO _4_, with average Kd values of ~100 mM even in water ( Table 1). On the other hand, shifts of the HSQC peaks of side-chain amide protons of WW4 could also be monitored upon titrating and subsequently their shift tracings were fitted to obtain apparent Kd values ( Table 1). Interestingly, only Na _2_SO _4_ appears to extensively interact to side-chain amide protons, in particular at pH 4.0. It is worthwhile to note that the Kd values for binding to the side-chain amide protons are approximately 3- to 4-fold larger than those of the backbone ones at the same condition, indicating that anions interact with backbone and side-chain amide protons separately.

**Table 1.** Apparent Dissociation Constants (Kd) for Binding of Salts to WW4 Domain ^a, b, c^.

Residue		Na _2_SO _4_			NaCl			NaSCN		Na _2_HPO _4_
					Backbone					
	pH 6.4	Buffered	pH 4.0	pH 6.4	Buffered	pH 4.0	pH 6.4	Buffered	pH 4.0	pH 6.4
**W9**		96.8 ±15.3					>250	>250		
**E10**							126.2 ± 24.4		81.8 ± 10.0	
**E16**							111.4 ± 20.1	>250	86.7 ± 5.5	79.7 ± 6.2
**G17**			18.0 ± 2.7				79.4 ± 18.3			
**V22**									116.7 ± 25.3	
**D23**		92.0 ± 11.4					135.4 ± 17.5	>250	95.0 ± 5.5	
**H24**								>250		
**N25**		76.6 ± 11.7			>250			>250	27.2 ± 3.2	
**T26**			12.6 ± 1.2						25.3 ± 1.5	
**R27**							115.0 ± 12.9		95.8 ± 13.1	
**F31**	30.3 ± 4.3	94.2 ± 6.6	18.9 ± 2.0					>250	150.7 ± 33.0	
**K32**	18.6 ± 2.8		13.3 ± 1.4				48.2 ± 8.6			
**R35**				>250			>250	>250		
**N36**	47.2 ± 4.1	71.9 ± 8.1		94.8 ± 13.9			82.0 ± 12.6	>250		149.1 ± 13.3
**Average ^b^**	**32.0**	**86.3**	**15.7**	**94.8**			**99.7**		**84.9**	**114.4**
					**Side chain**					
**N1s**			62.5 ± 6.5							
**N25s**	77.4 ± 16.8		38.5 ± 3.5				114.8 ± 17.3	>250	61.0 ± 11.7	
**N36s**	150.8 ± 28.4	>250	56.3 ± 5.5							
**R15s**			44.5 ± 7.9							
**R27s**			55.2 ± 6.1							
**R35s**			30.2 ± 3.3						98.6 ± 12.3	
**Average ^b^**	**114.1**		**47.9**				**114.8**		**79.8**	

^a^“Buffered” refers to “in 20 mM sodium phosphate (pH 6.4)”.

^b^Kd values are in mM;

^c^In calculating the average values, Kd values >250 mM are not included.

### Pre-existence of buffer masks the specific anion-binding

**Figure 21.**
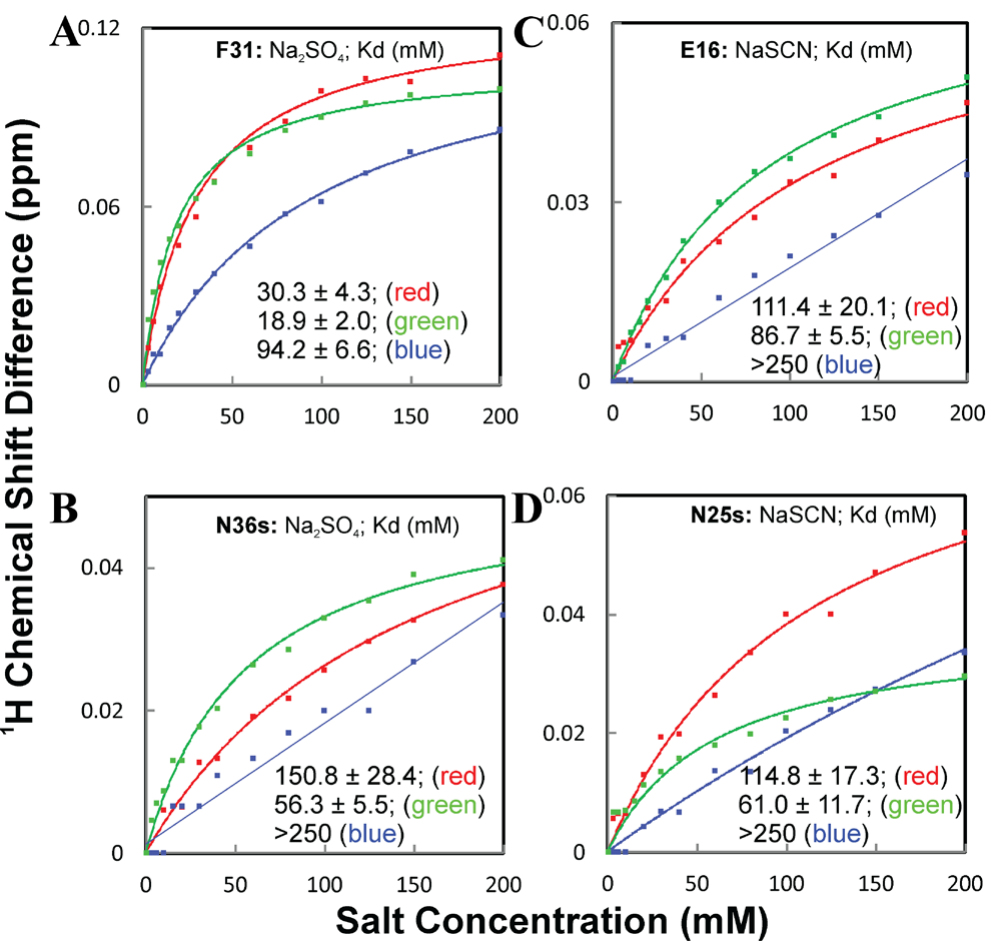
Apparent dissociation constants for representative amide protons of WW4. ( **A**)–( **B**) Residue-specific apparent dissociation constants (Kd) for backbone amide proton of Phe31 and side-chain amide proton of Asn36 titrated by Na _2_SO _4_ under different conditions. ( **C**)–( **D**) Residue-specific apparent dissociation constants (Kd) for backbone amide proton of Glu16 and side-chain amide proton of Asn25 titrated by NaSCN under different conditions. Experimental (dots) and fitted (lines) values are shown for the ^1^H chemical shift changes induced by gradual addition of two salts (Na _2_SO _4_ and NaSCN). Red is for the data in water (pH 6.4), green for those in water (pH 4.0), and blue for those in 20 mM sodium phosphate buffer (pH 6.4).

Strikingly, the pre-existence of 20 mM sodium phosphate buffer significantly reduces the binding affinity of all three salts, as exemplified by titration curves and Kd values of several representative residues ( Figure 21). For Na _2_SO _4_, although the presence of the buffer leads to an approximately 3-fold affinity reduction for backbone amide protons, titration curves still show saturation to some degree ( Figure 21A and Table 1). However, for NaSCN, the presence of the buffer renders the titration curves to appear to be almost linear which thus could not be fitted with good confidence ( Figure 21C and Table 1). For the side chain amide protons, the presence of the buffer leads the titration curves by both Na _2_SO _4_, and NaSCN to become completely linear ( Figure 21B and 21D).

**Figure 22.**
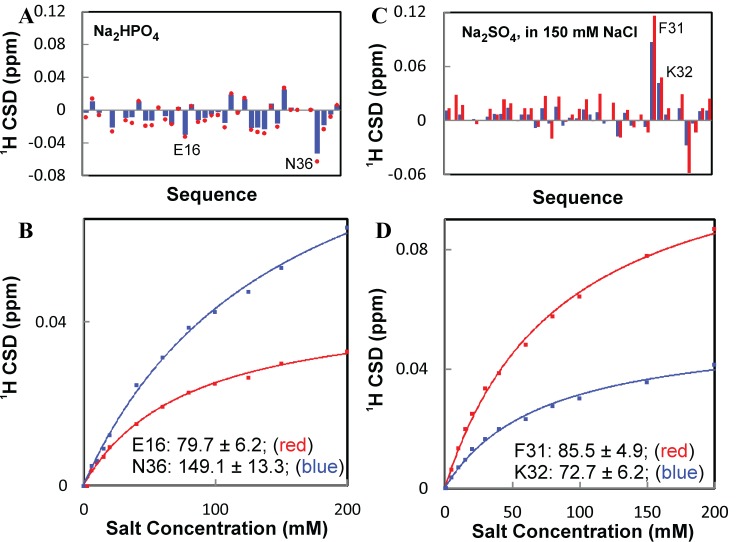
Effects of Na _2_HPO _4_ and NaCl in masking the specific anion-binding. ( **A**) Residue-specific chemical shift differences of amide protons ( ^1^H) of the WW4 domain upon addition of Na _2_HPO _4_ at 150 mM (blue bars) and 200 mM (red circles). ( **B**) Residue-specific apparent dissociation constants (Kd) for Glu16 and Lys32. Experimental (dots) and fitted (lines) values are shown for the ^1^H chemical shift changes induced by gradual addition of Na _2_HPO _4_. ( **C**) Residue-specific chemical shift differences of amide protons ( ^1^H) upon addition of Na _2_SO _4_ at 200 mM to the WW4 sample in unsalted water at pH 6.4 (red bars) and in unsalted water with the pre-existence of 150 mM NaCl at pH 6.4 (blue bars). ( **D**) Residue-specific apparent dissociation constants (Kd) for Phe31 and Lys32. Experimental (dots) and fitted (lines) values are shown for the ^1^H chemical shift changes induced by gradual addition of Na _2_SO _4_.

Strikingly, the pre-existence of 20 mM sodium phosphate also considerably changes the shift patterns by all three salts. In the buffer, some residues, which are not perturbed either by that salt or sodium phosphate separately ( Figure 22A), suddenly appeared to be significantly perturbed ( Figure 20). For example, in the phosphate buffer, Na _2_SO _4_ is suddenly able to significantly perturb Trp9, Asp23 and Asn25 ( Figure 20B), which are not largely perturbed by Na _2_SO _4_ alone in water either at pH 6.4 or 4.0 ( Figure 20A–C).

To understand the effect of the phosphate buffer, we titrated the WW4 domain with Na _2_HPO _4_ and the result showed that it only weakly binds to WW4, with only two amide protons having significant shifts of HSQC peaks ( Figure 22A) and subsequent fitting give rise to their apparent Kd value similar to those by NaSCN ( Figure 22B and Table 1). To assess whether the observed change of shift patterns is completely due to the non-specific electrostatic screening imposed by the presence of 20 mM sodium phosphate, we also titrated Na _2_SO _4_ to a WW4 sample in the pre-existence of 150 mM NaCl, which is considered to be the physiological concentration in blood and has an ionic strength larger than that of 20 mM sodium phosphate. Very unexpectedly, as shown in Figure 22C, the presence of 150 mM NaCl appears to largely attenuate the shift amplitudes but lead to no significant change in shift patterns. Noticeably, the presence of 150 mM NaCl does lead to ~3-fold reduction of the binding affinity of Na _2_SO _4_, to Phe31 and Lys32 ( Figure 22D).

### Diverse binding properties by different salts

Na _2_SO _4_, NaCl and Na _2_HPO _4_ appear to bind only a subset of well-exposed amide protons which are located on loops/turns or a short 3 _10_-helix over Pro34-Arg35-Asn36 ( Figure 19D). By a sharp contrast, NaSCN is even able to bind well-protected amide protons such as from Trp9, Glu10, Val22 and Asp23, which are on two central β-strands. In particular, Glu10 is one of two residues with the most protected amide protons (another is Ile11) with a Kex of 0.97 h ^-1^. Further analysis of electrostatic potential surfaces reveals a surprising picture: the amide protons interacting with Na _2_SO _4_, NaCl and Na _2_HPO _4_ are almost all located on the electrostatically positive regions while NaSCN is also able to bind to the amide protons of Trp9, Glu10, Val22 and Asp23 located on electrostatically negative regions ( Figure 19E-F). This implies that Na _2_SO _4_ binding is highly electrostatically driven while NaSCN is not. Indeed, as shown in Table 1, the binding affinities of Na _2_SO _4_ at pH 4.0 have a ~2-fold increase as compared to those at pH 6.4 while no significant difference is found for NaSCN at two pH values. Remarkably, the presence of 20 mM sodium phosphate renders Na _2_SO _4_ to behave like NaSCN, capable of significantly interacting Trp9 and Asp23 on two core β-strands with well protected amide protons ( Figure 19E and Figure 20B), while those two residues are not significantly perturbed by sodium phosphate alone even at a concentration up to 200 mM ( Figure 22A).

### Summary

By use of the last 33-residue subdomain as a reporter, we again demonstrate that protein conformations have no fundamental difference in unsalted water and buffer. Remarkably, our systematic study with 14 salts with 8 anions located differently in the Hofmeister series clearly indicates that the absence of the unique folded tertiary structures in unrefoldable and insoluble proteins solubilized in unsalted water is the manifestation of their intrinsic properties, not due to the absence of salt ions.

Surprisingly, we reveal that if we remove the interference of the pre-existing ions, 8 anions are all able to bind distinctive sets of ephrin-B2 residues with very high affinity. This selective and high-affinity binding by anions was further confirmed on a well-folded WW domain, despite having a ~10-fold reduction of the affinity. Markedly, sulfate anion is the tightest binder to both unstructured and well-folded proteins. The asymmetric anion binding appears to be modulated by three major factors: anion type, protein conformation and surface electrostatic potential. Highly hydrated sulfate, phosphate and chloride anions appear to only bind well-exposed amide protons, mostly driven by electrostatic interactions. The highest charge-density of sulfate anion appears to be the key factor responsible for the tightest interactions with amide protons. By contrast, thiocyanate is able to bind the largest set of amide protons including some well-protected amide protons located on electrostatically negative patches. This observation implies a fundamental difference between thiocyanate and other three anions. Indeed, thiocyanate was characterized to be weakly solvated with low charge density and consequently it seems that van der Waals interactions play a key role.

The scenario we uncovered is completely inconsistent with the current belief that protein-ion interactions are predominantly non-specific, electrostatic interactions at physiologically relevant ion concentrations (<100 mM) ^101^. It seems that the previous failure to observe selective anion binding to proteins with high affinity is most likely due to the pre-existence of buffer ions. Indeed, we have shown that the pre-existence of 20 mM sodium phosphate exactly as previously used ^101^ not only reduces the binding affinities of the anion-binding, but also alters the binding patterns, which is not observed in the pre-existence of 150 mM NaCl. Now we have obtained NMR relaxation evidence that different salts have very diverse effects on protein dynamics, particularly on µs-ms time scale and this effect is dependent of both cations and anions.

Our results also reveal that a well-folded protein is significantly shielded from anion binding. This result also implies that salt ions also play a key role in provoking misfolding for some protein mutants such as the T46I-MSP domain, which still possesses the capacity to fold into the native MSP structure, and only has cooperative unfolding lost and protein dynamics increased ^102^. The mutation-causing increase of protein dynamics may allow salt ions to more easily access the core region of the T46I mutant, which results in tighter anion-binding, and/or further changes of protein dynamics. All alterations together result in an enhanced misfolding of the mutant in the macromolecular crowded cells ^102, 103^.

## Transformation from non-membrane to membrane proteins, and emergence of primitive membranes embedded with integral membrane proteins

### Transformation from non-membrane to membrane proteins

The basic building block of all organisms is the cell, which is generally regarded as a space with a watery interior separated from the external environment by two layers of phospholipid molecules called the plasma membrane. Eukaryotic cells also have extensive internal membranes that further subdivide the cellular space into various compartments. However, the middle of membranes is constituted by fatty acid hydrocarbon tails, which are highly hydrophobic. Therefore, cellular membrane systems appear not only to build up a barrier for preventing the free flow of molecules in and out of cells, as well as among different cellular compartments, but also provide a hydrophobic phase, in particular in eukaryotic cells. As such, two phases with opposite polarity properties already coexist in cells, one is watery polar while another is membrane-based hydrophobic phases. The membrane-based hydrophobic phase may play many unrecognized but extremely important roles as the transition from prokaryotic to eukaryotic cells is associated with a dramatic emergence of large internal membrane systems.

As our studies on various insoluble proteins suggest that protein insolubility/aggregation mostly results from the unfavorable interaction between polar water molecules and hydrophobic side chains ^26^, it is thus tempting to speculate that insoluble proteins may have a stronger capacity to interact with membranes containing a hydrophobic phase in the middle. With our discovery as a powerful tool, we have tested this possibility by solubilizing insoluble proteins in unsalted water followed by gradual adding the lipid mimetic dodecylphosphocholine (DPC). The results show that the insoluble proteins tested are indeed all able to interact with lipids to different degrees.

**Figure 23.**
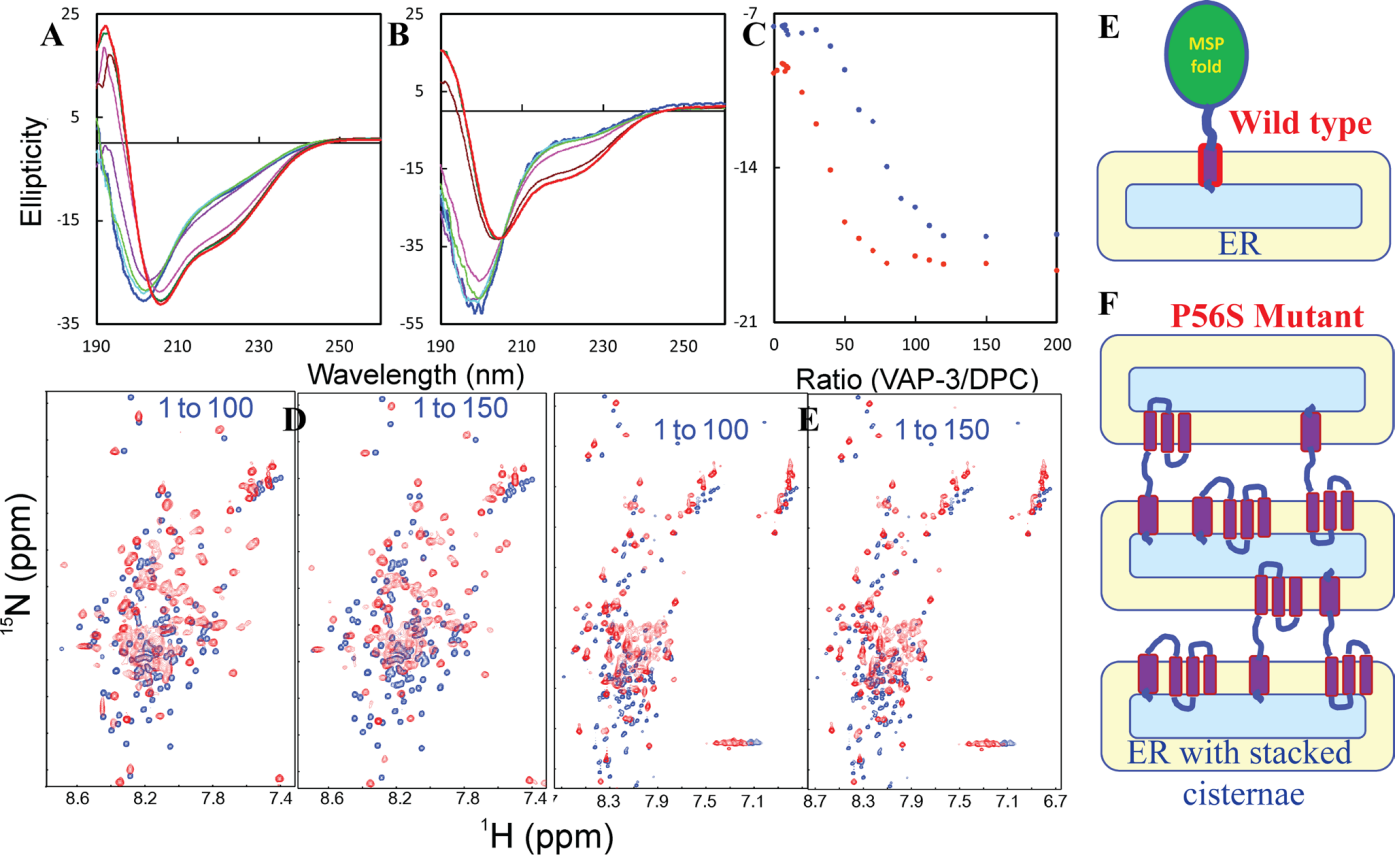
Transformation of the P56S-MSP and VAPB-3 into integral membrane proteins. ( **A**)–( **B**) Far-UV CD spectra of P56S-MSP ( **A**) and VAPB-3 ( **B**) in the absence of (blue) and in the presence of DPC at different ratios (protein:DPC): 1:6 (cyan), 1:10 (bright green), 1:20 (purple), 1:50 (pink), 1:100 (brown), 1:150 (green) and 1:200 (red). ( **C**) Ellipticity values at 222 nm of P56S-MSP (red) and VAPB-3 (blue) vs. the ratios (protein:DPC). ( **D**)–( **E**) ^1^H– ^15^N NMR HSQC spectra of P56S-MSP ( **D**) and VAPB-3 ( **E**) in the absence of (blue) and presence of DPC at two ratios (red) as indicated (P56S:DPC). ( **E**) Cartoon model of the wild-type VAPB protein anchored onto ER membrane, with the well-folded MSP domain in the cytosol. ( **F**) Cartoon model proposed here to rationalize a recent report that the P56S VAPB protein triggers the formation of a novel form of organized smooth endoplasmic reticulum with stacked cisternae. Briefly, the P56S-MSP domain becomes insoluble in the cytosol and thus will have a strong preference to insert into ER cisternae. If the C-terminal transmembrane fragment and P56S-MSP insert into different ER cisternae, a novel form of organized smooth ER will form with stacked cisternae.

In particular, we found that the ALS-causing P56S-MSP mutant and VAPB-3 are able to transform into well-formed helical structures with most residues buried in a membrane environment ^84^. The wild-type VAPB-MSP adopts a well-folded β-sandwich structure which is highly soluble in buffer ( Figure 12A) and shows no detectable interaction with DPC ^84^. By contrast, both P56S mutant ( Figure 23A) and VAPB-3 variant ( Figure 23B) are completely buffer insoluble, and are predominantly unstructured in unsalted water. However, both of them are able to interact with DPC and gradually transform into a highly helical structure ( Figure 23). As monitored by HSQC spectroscopy, the transformation is involved in almost all residues of P56S and VAPB-3. Once fully formed, the helical conformations account for 68% for both P56S and VAPB-3 and most residues appear to be buried in the DPC micelle. This suggests that for P56S-MSP and VAPB-3, it is thermodynamically more favorable to insert into membranes to form high helical state than to stay in unsalted water as a predominant disordered state.

Our results provide a mechanism to rationalize a recent report that the P56S mutant is able to trigger the formation of a novel form of organized smooth endoplasmic reticulum (ER) with stacked cisternae ^104, 105^, despite failing to detect significant formation of aggregated inclusions in motor neurons derived from induced pluripotent stem cells of patients carrying the P56S mutation ^106^. In light of our discovery, the formation of ER with stacked cisternae can be nicely explained. Briefly, as shown in Figure 23E, the wild-type VAPB protein is anchored onto the ER by the C-terminal transmembrane fragment, and its well-folded MSP domain is in the cytosol. However, on the advent of the P56S mutation, the mutated MSP domain becomes completely insoluble in the cytosol and will spontaneously insert into ER membranes: some into the same cisternae, some into the different cisternae ( Figure 23F). The insertion into different cisternae will lead to the formation of an ER with stacked cisternae, which has been observed in cells ^104, 105^. Furthermore, if the P56S mutant protein is over-expressed in cells, the membrane systems may not be able to accommodate all of it, and so it gets accumulated as visible inclusions/aggregates.

Our results thus demonstrate that many insoluble proteins are potentially able to interact with membranes, and non-membrane proteins can easily transform into membrane-interacting proteins by slight mutations/truncations/modifications. However such membrane-interacting proteins cannot be detected by current bioinformatics tools and consequently the number of membrane-interacting proteins may be much larger in cells than currently recognized. Also attacking membrane systems before the accumulation of visible aggregates/inclusion may represent a general mechanism by which insoluble proteins trigger human diseases such as neurodegenerative diseases. Our results also imply a potential mechanism to connect sporadic and familiar human diseases. For example, the fact that VAPB-3 shares similar properties with P56S-MSP as regards membrane interactions, means that even without carrying any ALS-causative mutation, a person might develop ALS through a mechanism underlying ALS8, if VAPB slicing variants like VAPB-3 get accumulated in cells to a certain degree, due to proteasomal inhibition triggered by pathological and/or environmental conditions. Indeed, the VAPB-3 protein was found to become detectable in cells only upon proteasomal inhibition, a condition commonly found in all neurodegenerative diseases ^85^.

### Emergence of primitive membranes embedded with integral membrane proteins

In a TIBS article ^107^, Mulkidjanian and colleagues found it enigmatic how the integral membrane proteins could reach the primeval membranes, since these proteins, having extremely high hydrophobicity, are water-insoluble and consequently "even if occasionally synthesized, would remain stuck in the ribosome" ^107^. As a consequence, emergence of protein-embedded primeval membranes is called a 'chicken and egg paradox'. However, in light of our discovery, this paradox can be solved as some evidence suggests that proteins and primitive membranes with integral membrane proteins might emerge in unsalted oceans with a slightly acidic pH ^107, 108^. This prebiotic condition is amazingly very similar to what my lab commonly used to solubilize insoluble/membrane proteins. Consequently, as supported from our results, even the most hydrophobic integral membrane peptide would not be stuck in the ribosome in unsalted oceans. Instead, they would be certainly soluble in such a prebiotic unsalted medium. In particular, the concentrations for most, if not all, proteins might also be very diluted in the primitive oceans and consequently they would be able to freely diffuse around to reach the primeval membranes to achieve spontaneous assembly, as shown for P56S-MSP and VAPB-3.

Our discovery might also shed light on another mysterious issue associated with the diversification of proteins. It was proposed that the space of realized protein folds appears to just account for one-tenth of the space of possible folds ^109^. This implies that a large portion of the sequence space remains unexplored in the life forms on Earth. Here I hypothesize that this might be associated with ocean salinity. The machinery generating proteins is believed to have existed before the emergence of the membrane-enveloped primitive cells ^109, 110^. In unsalted oceans and without membranes, proteins created with their sequences highly randomized were all soluble and thus could diffuse freely to encounter other proteins, other biomacromolecules, organic and inorganic small molecules for self-assembly into various complexes/machineries. The slight increase in salt concentrations in oceans might facilitate the assembly of some protein-based complexes or machineries. Indeed, we frequently observed that in unsalted water, protein-protein interactions are dramatically suppressed due to the repulsive electrostatic interactions between individual protein molecules. The presence of salt ions is expected to reduce repulsive electrostatic interactions by a non-specific screening effect and/or specific anion-binding, thus allowing protein-protein, and other, interactions.

On the other hand, once the membrane-contained cells were formed and the oceans became highly salted, proteins with high hydrophobicity would become aggregated and/or start to attack membranes. This might trigger the emergence of mechanisms to halt the high randomized sampling of the sequence spaces. In this regard, all modern proteins regardless of being well-folded; intrinsical unstructured and membrane-associated might be all diverged from the constrained numbers of the primordial proteins which were randomly created in unsalted oceans. This scenario perfectly rationalizes my previous proposal that "[modern] proteins appear so designed that in pure water their intrinsic repulsive interactions are sufficient to suppress the attractive forces, thus preventing them from severe precipitation/aggregation" ^26, 68^. It might also be possible that the current 20 natural α-amino acids in the L-image were selected because their polymerized products, proteins, were all soluble in the primitive unsalted oceans.

## Conclusion remarks and further directions

### Birth, transformation and death of proteins

**Figure 24.**
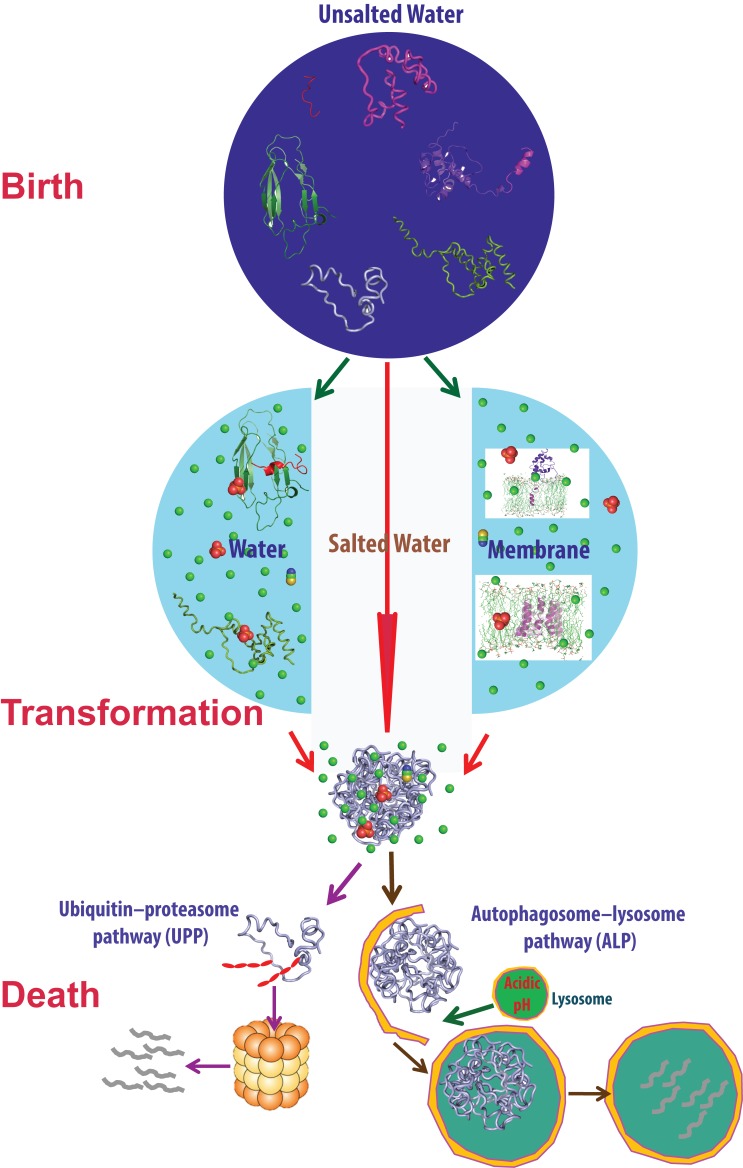
Birth, transformation and death of proteins. In the unsalted water, which may mimic the prebiotic medium where proteins originally emerged, all proteins, regardless of their hydrophobicity, being well-folded or unstructured, are all soluble and being able to diffuse freely. However, upon becoming salted, only well-folded proteins and a portion of intrinsically unstructured proteins (IUPs) remain soluble in salted aqueous solution. The salted conditions may facilitate the formation of various protein-based complexes such as among well-folded proteins, between well-folded protein and unstructured peptides/proteins; and among unstructured peptides/proteins. On the other hand, upon encountering lipid molecules, some proteins such as those with high hydrophobicity may transform into membrane proteins. Nevertheless, there might be a portion of proteins designated as 'intrinsically insoluble proteins (IIPs)', which completely lack the intrinsic capacity to fold into any well-defined structures and also have their hydrophobic patches improperly exposed to aqueous bulky solution. For certain reasons, these proteins fail to reach and/or cannot be completely accommodated by biological membrane systems. Aggregation is consequently their evitable destination *in vivo* with ~150 mM ion concentration. As the aggregation of IIPs cannot be recovered by cellular chaperone systems, to minimize their harmful effects eukaryotic cells have developed many complex degradation machineries to degrade IIPs and 'misfolded proteins', which include ubiquitin–proteasome pathway (UPP) and autophagosome–lysosome pathway (ALP).

In the past several years, our discovery has provided a powerful tool for us and other groups to characterize a variety of unrefoldable and insoluble proteins. So far, my lab has encountered ~60 such insoluble proteins but found that all of them could be solubilized in unsalted water for high-resolution biophysical studies, some of which have been published ^22, 67– 69, 84, 91, 93, 96, 111– 113^. Most strikingly, we demonstrated that the 25-residue M2 transmembrane peptide of influenza A proton channel, one of the most hydrophobic sequences in nature, could also be solubilized in unsalted water without lipid molecules to form a highly helical conformation. This implies that only in unsalted water are all proteins are able to manifest their intrinsic conformations, regardless of their hydrophobicity and whether they are well-folded, partial folded or predominantly unstructured ( Figure 24). So why are all proteins soluble in unsalted water? One scenario that rationalizes this phenomenon is that the prebiotic aqueous medium where proteins originally emerged was largely unsalted. The current 20 α-amino acids in the L-image conformation were selected because their products, proteins, are all soluble in such a medium. Indeed, there exists evidence indicating that when primitive proteins were made, the ocean was highly unsalted and slightly acidic ^107, 108^. Moreover, it has been shown that the presence of ions even at concentrations much lower than those of contemporary oceans would impose adverse effects on membrane self-assembly and RNA polymerization, and consequently the prebiotic medium could not have been salted ^114^.

Amazingly, only this scenario can provide a solution to the 'chicken and egg paradox' for the origin of the primeval membranes embedded with integral membrane proteins. As the most hydrophobic integral membrane peptide is soluble in unsalted water, all primitive membrane proteins regardless of their hydrophobicity are anticipated to be soluble in unsalted prebiotic oceans and to diffuse around freely. Upon encountering primitive membranes, these proteins would insert into the hydrophobic phase of the membranes and transform into membrane proteins ( Figure 24). This transformation process appears to be thermodynamically favorable as implied from our result that the unstructured VAPB-3 and P56S-MSP domain can spontaneously insert into membranes in unsalted water and transform into high helical structures. The transformation into membrane proteins might also serve to protect proteins with high hydrophobicity from becoming aggregated upon the later increase in salt concentrations. Consequently protein aggregation might not be a severe problem in primitive cells even with high salt concentrations. It appears that even in prokaryotic cells, under normal physiological conditions, protein aggregation is mainly involved in 'off-pathway' misfolding of proteins which can assume well-folded structures and are soluble in the salted cytosol, rather than 'intrinsically insoluble proteins'. Indeed, the presence of molecular chaperone systems plus minor degradation activity appears to be sufficient to minimize the accumulation of aggregated proteins. Consequently, complex protein degradation machineries have been underdeveloped in prokaryotic cells.

In contrast, eukaryotic cells appear to face a serious problem of protein aggregation even at unstressed conditions. Very surprisingly, based on results with human cell lines, it has been estimated that ~30% of newly synthesized proteins get aggregated and rapidly degraded by proteasomes ^58, 115, 116^. These proteins were called as defective ribosomal products (DRiPs), "that never attain native structure owing to errors in translation or post-translational processes necessary for proper protein folding" ^58^, but their exact nature remains to be defined. Here I propose that the 'intrinsically insoluble proteins (IIPs)' we establish here may account for a large portion of DRiPs. As implied from the results with 'intrinsically insoluble proteins' *in vitro*, the aggregation might be also an inevitable destination for them *in vivo* due to their lack of the intrinsic capacity to fold into unique tertiary structures and high ion concentrations *in vivo*. In particular, unlike 'misfolded proteins', the aggregation of 'intrinsically insoluble proteins' cannot be overcome by chaperone systems. Therefore, despite being an immense waste, the only option for cells to minimize their harmful effects is to remove them by degradation immediately after their synthesis ^58, 115, 116^.

The emergence of a large amount of 'intrinsically insoluble proteins' in eukaryotic cells appears to be associated with at least three well-known characteristics of eukaryotic genomes: 1) increase of intrinsically unstructured proteins with low-complexity sequences; 2) emergence of the splicing variation; and 3) accumulation of random mutations. The underlying mechanisms for generating 'intrinsically insoluble proteins' by these three processes can be exemplified by our results with three naturally occurring insoluble proteins: ApLLP, a full-length wild-type protein with a low complexity sequence; VAPB-3, a splicing variant with truncation on the well-folded MSP domain; and P56S-MSP, a ALS-causing mutant with only a single-residue mutation on the MSP domain. On the other hand, as we frequently observed, the interactions responsible for protein aggregation are also important for the folding and interactions. Therefore, nature may or may not be able to optimize protein sequences to completely avoid aggregation. As a result, eukaryotic genomes are abundant in 'intrinsically insoluble proteins' and, to cope with them, eukaryotic cells have developed many complex machineries to remove them, which include the ubiquitin–proteasome pathway (UPP) and the autophagosome–lysosome pathway (ALP) ( Figure 24). Indeed, proteins involved in proteasomes have been found to account for 1% of total cellular protein ^58^. Degradation of various aggregated proteins might represent a central mission for eukaryotic cells to survive and the failure of the degradation systems might have catastrophic consequences for the organisms, which include various aggregation-causing diseases and aging.

Wonderfully, a cell is already composed of two opposite, but complementary phases in terms of polarity. While cellular compartments contain the polar watery phase, cellular membrane systems provide the hydrophobic hydrocarbon phase. As we have shown, insoluble proteins have a preference to interact with membranes to different degrees. This implies that insoluble proteins may have potential to transform into membrane-interacting proteins. Indeed, as we recently uncovered, upon encountering lipids the insoluble VAPB-3 and P56S-MSP mutant transform into integral membrane proteins with the majority of residues buried in the membrane environment. Therefore, it is tempting to hypothesize that biological membranes emerge in evolution not only to separate the interior space of a cell from environments, and to achieve compartmentalization within eukaryotic cells, but also serve to provide a hydrophobic phase to host proteins with high hydrophobicity. It is possible that the emergence of large internal membrane systems in eukaryotic cells might also represent part of cellular response to the dramatic increase of 'intrinsically insoluble proteins' in eukaryotic genomes. In other words, the emergence of internal membrane systems may help accommodate many insoluble proteins by facilitating their transformation into membrane proteins, and, mutually, these newly formed membrane proteins might also contribute to the formation and maintenance of novel structures of the internal membrane systems. Indeed, it has been extensively found that over-expression of membrane proteins would trigger expansion and structural changes of eukaryotic internal membranes and, in particular, it even resulted in the formation of internal systems in *E. coli* cells ^117– 121^. Moreover, ER membrane structures and dynamics have been demonstrated to be critically regulated by the presence of various membrane-interacting proteins ^104, 105, 118– 121^.

Theoretically, the existence of both watery and membrane phases in cells would be sufficient to accommodate proteins with various hydrophobicity. Nevertheless, in modern cells with ~150 mM ion concentration, without the specific assistance by complex machineries such as translocon ^31^, most 'intrinsically insoluble proteins' would be stuck in ribosomes before they are able to reach membranes. Consequently they will be either degraded immediately after they are synthesized, or accumulated upon over-expression and/or inhibition of the degradation machineries triggered by pathological conditions. The presence of ~150 mM ions may also represent a cellular mechanism to prevent the insoluble proteins to access membranes. Nevertheless, a portion of such proteins, in particular those that are abnormally over-expressed, might still be able to reach membranes. Therefore, to attack biological membranes may represent a general mechanism by which over-expression/accumulation of insoluble proteins initiates various aggregation-causing diseases.

### Salt ions act as 'dark mediators'

All results together by us and other groups reveal a fact that unrefoldable and insoluble proteins, including the most hydrophobic integral membrane peptide, are all soluble in unsalted water, but become aggregated upon being exposed to salt ions. This logically suggests that salt ions play a role at least as important as proteins in mediating protein aggregation. As salt ions exist in the background and such key roles have been largely unrecognized, I thus designate them as 'dark mediators' as analogous to 'dark matter' ^92^. The factors from proteins and salt ions are equally important in modulating protein aggregation, which can be nicely symbolized by the Taji diagram ( Figure 6).

The most frequently asked question, when I presented speeches on our discovery in conferences, is whether the lack of the well-defined structures observed on unrefoldable and insoluble proteins solubilized in unsalted water is due to the absence of salt ions. To systematically address this question, we have selected the insoluble cytoplasmic domain of ephrin-B2 as a model system and titrated it with 14 salts with 8 anions located in the middle, on the left and right sides of Hofmeister series. This set of results, together with those we previously obtained by titrating unrefoldable and insoluble proteins with NaCl, clearly demonstrate that the solution conformations have no fundamental difference in unsalted and salted water. Therefore, the lack of tight tertiary packing has been confirmed to represent an intrinsic feature associated with unrefoldable and insoluble proteins, as I have previously proposed ^26, 68^.

Unexpectedly, however, our studies unveil that in contrast to the common belief, anions are able to asymmetrically bind both unstructured and well folded proteins with high selectivity and affinity at physiological relevant concentration (<200 mM). The anion binding has been characterized to be mediated by anion type, protein conformation and surface electrostatic potential. Remarkably, the well-folded protein is significantly shielded from anion binding in terms of the number of binding sites and affinity. Surprisingly, the selective anion binding with high-affinity can be dramatically masked by the pre-existence of salt ions. Intriguingly, the pre-existence of 20 mM sodium phosphate not only masks the selective anion binding but also alters the binding patterns. Now we have obtained NMR relaxation data revealing that this is mostly due to very diverse effects by different salts on protein dynamics, particularly on µs-ms time scale.

**Figure 25.**
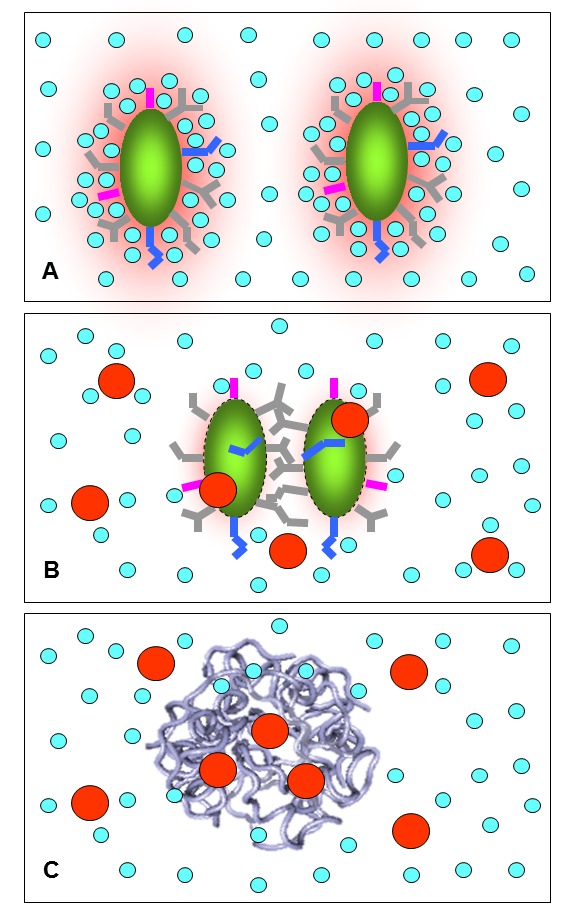
Modified model to rationalize how salt ions mediate protein aggregation. ( **A**) An unrefoldable and insoluble protein in unsalted water with the solution pH several unites away from its pI. Small cyan spheres stand for water molecules and green ellipsoids for protein molecules with a large amount of hydrophobic side chains exposed. ( **B**) Protein molecules in the presence of a small amount of salt ions (larger red spheres). In addition to imposing non-specific electrostatic screening as I previously proposed, the presence of salts also provides specific anion binding to protein residues, thus altering the surface electrostatic potential. Furthermore, salts may also changes water clustering structure as well as protein dynamics as represented by the broken lines of green ellipsoids. ( **C**) The complex interplay of these salt effects may result in aggregation of the protein.

In 2008, I proposed a model to rationalize why unrefoldable and insoluble proteins can be soluble in unsalted water, but become aggregated upon introduction of salt ions even at very low concentration. However, in the previous model only the non-specific electrostatic screening effect is considered ^26^. Briefly, due to the lacking of the tight tertiary packing, these proteins have a substantial amount of hydrophobic side chains exposed to the bulk water. If a protein of this family is dissolved in unsalted water with pH deviated from its pI, the individual molecules will bear a significant amount of net charges ( Figure 25A). In this regard, the repulsive electrostatic interaction and/or large protein hydration shell will constitute an energy barrier unfavorable for inter-molecular interactions. Consequently, in unsalted water, protein aggregation is significantly suppressed. However, even if a small amount of salt ions is introduced, the repulsive electrostatic interaction will be screen out and/or the protein hydration shell may be disrupted to some extent. As a sum, the hydrophobic interaction will become dominant, thus leading to immediate aggregation ( Figure 25C).

Here, based on our new results, I modify the model to include both specific anion binding and salts’ effect on protein dynamics ( Figure 25B). The presence of the two specific effects appears to suddenly complicate the relationship between ion type/concentration and protein solubility. Upon introducing salt ions, the reduction of repulsive electrostatic interactions by non-specific screening may still represent a major consequence. On the other hand, the specific anion binding can either result in neutralizing the net charges, or leads to the introduction of extra charges. For example, a protein with a neutral pI will have a positive net charge in acidic solutions. The binding by mono-valent anions such as chloride only to the positively charged regions of the protein is expected to neutralize the positive charges, thus leading to further reduction of repulsive electrostatic interactions. Nevertheless, the sign of the net charge may be reversed if this protein has a large number of positively charged patches which are tightly bound by multi-valent anions such as sulfate; or both hydrophobic and hydrophilic patches of this protein are extensively bound by anions such as thiocyanate. For such special situations, the relationship between ion concentration and protein solubility could be extremely complex. Here it is tempting to hypothesize that in human fluids, chloride has been selected as the dominant anion in evolution, at least partly due to its minimal ability in binding to proteins and in altering protein dynamics.

Electrostatic interactions have been recently proposed to play key roles in modulating protein structures, interactions and assemblies in cellular crowding environments ^122^. The specific but extremely complex effects we found for salt ions also on well-folded proteins imply that in addition to their central roles in mediating protein aggregation, salt ions may also act to modulate various biological functions of proteins. The variation of salt types and concentrations in cells may have significant impacts on proteins, including aggregation, which are ultimately associated with human diseases. Therefore, it would be critical to decipher their molecular mechanisms by experimental and computational approaches for establishing therapeutic strategies. On the other hand, the existence of specific anion-binding also implies a marked challenge to simulate protein-protein, protein-ligand interactions due to the current difficulty in computationally assessing specific interactions between proteins and anions.

### Future directions

Over the past few years, our discovery has allowed extensive characterizations by us and other groups on unrefoldable and insoluble proteins, which not has elucidated previously unknown regimes associated with proteins; but has also provided critical insights into aggregation-causing diseases. On the other hand, fundamental questions have arisen which can be grouped into the following four categories.

First of all, protein aggregation in watery media is most likely to result from the unfavorable interactions between polar water molecules and hydrophobic side chains of proteins. However, the relationship between hydrophobicity of a protein sequence and its solubility seems extremely complex. This is even true for the intrinsically unstructured ApLLP. ApLLP has a low complexity sequence and low hydrophobicity (~30%). However, it is completely insoluble in buffer. More intriguingly, removal of the N- or C-terminal fragment to give a slightly lower hydrophobicity could result in buffer soluble forms ^22^. Also, while the P56S mutant and splicing variant of the MSP domain are completely buffer-insoluble and predominantly disordered in unsalted water, another splicing variant VAPC is buffer-soluble with a disordered conformation in buffer similar to those of P56S-MSP and VAPB-3 in unsalted water ^80^. Therefore, it is of fundamental interest to delineate whether this complexity is just due to the context-dependent nature for protein aggregation, or as recently proposed, is due to its non-Euclidian landscape. If so, the prediction of protein aggregation from sequences is not just technically challenging, but is non-deterministic polynomial time (NP) complete ^87^.

Secondly, previously it has been well-established that many well-folded proteins can misfold into aggregated/amyloid forms to trigger human diseases, due to their dynamic nature and co-existence of many partial folded intermediates induced by slight changes of solution conditions ^45, 46, 102, 123– 125^. Here, we establish the existence of 'intrinsically insoluble proteins (IIPs)', some of which also cause neurodegenerative diseases such as ALS. The concept of 'IIPs' is in a nice agreement with the recent observation that ~30% of cellular proteins are DRiPs. Therefore, in the future, it is of particular essence to experimentally characterize the conformations of DRiPs by solubilizing them in unsalted water. This study will offer an estimation of the percentages of 'misfolded proteins' and 'intrinsically insoluble proteins' in DRiPs. This knowledge is crucial for future development of therapeutic strategies for treating aggregation-causing human diseases. To treat misfolding-triggered diseases, the effort may be devoted to preventing 'off-pathway' misfolding and/or to recover misfolded proteins, such as by enhancing the chaperone function. By contrast, to deal with 'IIPs'-causing diseases, the major focus might be to warrant the normal function of degradation machineries.

Thirdly, now it has been increasingly recognized that the chameleon transformation of protein conformations is not that uncommon. For example, the β-barrel structure of the hNck2 SH3 domain could transform into a similar helical conformation triggered by either acid-induced unfolding or mutation to disrupt tertiary interactions ^93, 95, 126^. However, it is still surprising to discover the transformation of the unstructured P56S-MSP domain in unsalted water into a well-folded helical structure in a membrane environment, as its hydrophobicity is much lower than that expected for a membrane protein. This implies that becoming a membrane is not highly dependent on the sequence hydrophobicity. In this regard, the P56S-MSP domain represents a unique model for further deciphering the general principles which direct folding, stability and evolution of membrane proteins.

Finally, our *in vitro* studies and the *in vivo* identification of DRiPs accounting for 30% cellular proteins ^58^ implies that protein sequences have not been successfully optimized to avoid aggregations. Therefore, a fundamental question arises whether this failure is due to the overlap of interactions responsible for aggregation with those requested for folding and interaction, or whether in nature there might be no so-called optimization restrained by 'functions'. Living systems might only be a natural manifestation of principles of self-organization in terms of structures and dynamics of all biomolecules and surrounding components. 'Function' is nothing but just an interpretation of these structures and dynamics in the context of relevant interaction networks.
