# Supplementary material for: Why do proteins aggregate? “Intrinsically insoluble proteins” and “dark mediators” revealed by studies on “insoluble proteins” solubilized in pure water
Source: F1000Res. 2013 Mar 22;2:94. [Version 1] doi: 10.12688/f1000research.2-94.v1 (PMC3869494; doi:10.12688/f1000research.2-94.v1)
Supplement: Peer review history

# Peer review: Referee response for version 1

**Published:** 2013-06-24

**Authors:** Jonathan W Yewdell

**Affiliations:** 1 National Institute of Allergy and Infectious Diseases, Bethesda, MD, USA

I have read this submission. I believe that I have an appropriate level of expertise to confirm that it is of an acceptable scientific standard.

# Peer review: Referee response for version 1

**Published:** 2013-05-02

**Authors:** Monika Fuxreiter

**Affiliations:** 1 Department of Biochemistry and Molecular Biology, Medical and Health Science Center; University of Debrecen, Debrecan, Hungary

The abstract is fine but I think the title is somehow misleading. The review does not give an answer as to why proteins aggregate and how pathological aggregations can be reversed or prohibited. The title should be more specific and scientific (avoid ‘dark mediators’). 

- Article content: The self-assembly of proteins is a very important problem not only from a folding point of view, but also from evolutionary or pathological aspects. The proposal that pure water can prohibit or reverse aggregation is interesting. I do not feel the presented results are convincing enough to provide a deeper insight into the mechanism of the aggregation phenomenon. 
- Conclusions are made based on a few examples and miss robust statistical tests on larger databases. For example, low-complexity sequences that are unstructured and insoluble, or proteins with high-complexity sequences that are unstructured and insoluble. Protein disorder is associated with a particular composition of amino acids, and has many other characteristics that are easy to detect. Intrinsically disordered (ID) proteins however are soluble, in many cases ID segments help to solubilize aggregation-prone regions. 
  The relationship between ID and aggregation is controversial. Aggregation-prone regions also have a particular composition (for example see R. Pomès *et al*., 2006  for differences between elastic and aggregating sequences). This review does not reflect the complexity of the theme and often runs into simplified conclusions in this respect.
- I would like to see the mechanism for how ions mediate aggregation and what the impact of their removal is. The relationship between ions and protein structure has been analyzed by various authors, and these works would be worth a mention (e.g. B. M. Pettitt *et al*., PMID: 19548651, PMID: 20151732, PMID: 20306490)
- The author argues for ‘specific binding’ of ions. It is obvious from various other works that ions bind at particular locations, but specificity has not been demonstrated clearly in this paper. Would ions make a difference between a WT protein and a single mutant?
- I think that the conclusions go beyond the scope and validity of the results, in particular ‘birth, transformation and death of proteins’.  This process is so complex and presented in an over-simplified manner here. How ID, which is identified here as one of the main factors, is related to degradation for example? 
- How can pure water reverse the effect of aggregation promoting ions? Just removing the ions does not guarantee that the protein will find its correct folding pathway. What about the hydrophobic patches in general? Will they still dislike water and try to avoid?
- I would strongly argue with the expression ‘dark mediators’. Ions are not dark. By neutron scattering and some modern SAXS data we can have detailed structural information on them.
- What would be the real practical application of the work?
- I feel the evolutionary arguments are rather speculative. There are some studies on ID evolution, maybe a good point to start with.

Overall, I feel the review – although many experimental data are presented – to be speculative and conclusions are not supported with robust analysis. The proposal is interesting but should be corroborated more. I also miss a general mechanistic picture, with thermodynamic and structural arguments given.

I have read this submission. I believe that I have an appropriate level of expertise to confirm that it is of an acceptable scientific standard, however I have significant reservations, as outlined above.

# Peer review: Referee response for version 1

**Published:** 2013-03-27

**Authors:** H Jane Dyson

**Affiliations:** 1 Department of Molecular Biology, The Scripps Research Institute, Jupiter, FL, USA

This is a very lengthy review on the contributions of dissolved ions to the behavior (aggregation, membrane interaction) of proteins. It is rather long and rambling (and seems somewhat repetitive) and may be construed to some extent as “propaganda” for the author’s opinion in this case. There also does seem to be quite a lot of primary data, ie, data appearing in this review that have not received peer review in other journals. However, on balance, the review is interesting, and should spark dialogue if not controversy in the scientific community.

I have read this submission. I believe that I have an appropriate level of expertise to confirm that it is of an acceptable scientific standard.
